# Selenization of *S. cerevisiae* increases its protective potential in experimental autoimmune encephalomyelitis by triggering an intestinal immunomodulatory loop

**DOI:** 10.1038/s41598-020-79102-7

**Published:** 2020-12-17

**Authors:** Thais Fernanda de Campos Fraga-Silva, Luiza Ayumi Nishiyama Mimura, Larissa Ragozo Cardoso de Oliveira, Juliana Helena dos Santos Toledo, Patrícia Aparecida Borim, Sofia Fernanda Gonçalvez Zorzella-Pezavento, Diego Peres Alonso, Paulo Eduardo Martins Ribolla, Carlos Alberto Ferreira de Oliveira, Denise Morais da Fonseca, Eduardo J. Villablanca, Alexandrina Sartori

**Affiliations:** 1 Botucatu Medical School, São Paulo State University (UNESP), Botucatu, Brazil; 2 Institute of Biosciences, São Paulo State University (UNESP), Botucatu, Brazil; 3 Institute of Biotechnology (IBTEC), São Paulo State University (UNESP), Botucatu, Brazil; 4 Biorigin, Zilor, Lençóis Paulista, Brazil; 5 Institute of Biomedical Sciences, University of São Paulo (USP), São Paulo, Brazil; 6 Immunology and Allergy Unit, Department of Medicine, Solna, Karolinska Institutet and University Hospital, Stockholm, Sweden

**Keywords:** Immunology, Autoimmunity, Mucosal immunology

## Abstract

Multiple sclerosis is an autoimmune disease that affects the myelinated central nervous system (CNS) neurons and triggers physical and cognitive disabilities. Conventional therapy is based on disease-modifying drugs that control disease severity but can also be deleterious. Complementary medicines have been adopted and evidence indicates that yeast supplements can improve symptoms mainly by modulating the immune response. In this investigation, we evaluated the therapeutic potential of *Saccharomyces cerevisiae* and its selenized derivative (Selemax) in experimental autoimmune encephalomyelitis (EAE). Female C57BL/6 mice submitted to EAE induction were orally supplemented with these yeasts by gavage from day 0 to day 14 after EAE induction. Both supplements determined significant reduction in clinical signs concomitantly with diminished Th1 immune response in CNS, increased proportion of Foxp3^+^ lymphocytes in inguinal and mesenteric lymph nodes and increased microbiota diversity. However, Selemax was more effective clinically and immunologically; it reduced disease prevalence more sharply, increased the proportion of CD103^+^ dendritic cells expressing high levels of PD-L1 in mesenteric lymph nodes and reduced the intestinal inflammatory process more strongly than *S. cerevisiae*. These results suggest a clear gut-brain axis modulation by selenized *S. cerevisiae* and suggest their inclusion in clinical trials.

## Introduction

Multiple sclerosis (MS) is considered the most common inflammatory and demyelinating disease of the central nervous system (CNS). Despite some controversy, there is a large body of evidence that MS is an autoimmune pathology, mainly mediated by specific immune response against the myelin sheath that wraps around nerve fibers^1^. T cell subsets, specially T helper (Th)-1 and Th17, cytotoxic T and innate cells as macrophages and microglia have been deeply implicated in MS pathobiology^2^. Details of MS immunopathogenesis have been revealed with the help of experimental autoimmune encephalomyelitis (EAE), which is an animal model induced mainly in mice by immunization with CNS antigens associated with complete Freund´s adjuvant and frequently with pertussis toxin^3^.

MS treatment is mostly based on the use of the so-called disease-modifying therapies (DMT). Currently approved DMT include interferon (IFN)-β, glatiramer acetate, mitoxantrone, natalizumab, fingolimod, teriflunomide, dimethyl fumarate and alemtuzumab^4^. In general, these drugs modulate or suppress different stages of the ongoing autoimmune response^5^. Despite their undeniable benefit, mainly in the relapsing–remitting MS, efficacy, safety, tolerability and compliance to treatment may vary widely among patients^6,7^. Severe side effects as cardiomyopathy, bradyarrhythmias, autoimmune thyroiditis and infections have been recorded^8,9^.

Considering the limitation of classical treatments, complementary and alternative medicines (CAM) have been suggested and some already adopted in MS therapy^10,11^. Roy Swank proposed a low saturated fat diet to treat MS around 1950^12–14^ and diet supplementation with organic, gluten-free and allergen-free ingredients have been beneficial to MS patients^15^. A review containing scientifically unproven data sustains the use of specific diets in the management of MS, suggesting a relationship between them and disease progression^16^. The immunomodulatory properties of CAM have been studied and their effectiveness in MS has been investigated. A relationship among diet, gut microbiota and MS has been demonstrated, reinforcing the presumption that prebiotic and probiotic supplementation could modulate the microbiome and autoimmunity in MS^17,18^. A great deal of knowledge is already established concerning how eubiosis/dysbiosis conditions are established^19^ and this allowed to select natural supplements with the ability to restore or modify the microbiota, amplifying therefore, the possibility to study probiotics as CAM.

In this context, bacterial and yeast probiotics have been tested in experimental and human MS. The oral administration of heat-killed *Candida kefyr*, for example, clearly decreased EAE severity. This protective effect occurred along with significant changes in intestinal immunity and microbiota as decreased Th17 cells, increased regulatory T and CD103^+^ dendritic cells (DC) and increased and decreased proportion of Lactobacillales and Bacteroides, respectively^20^. EAE neuroinflammation has also been controlled by the probiotic *Lactobacillus reuteri* DSM 17938^21^. *Saccharomyces cerevisiae*, which is considered a safe probiotic, has also been effective in controlling experimental vaginal candidiasis^22^ and enterotoxigenic *Escherichia coli* infection^23^. Even though *S. cerevisiae* was not directly tested in EAE, supplementation with molecules and fractions derived from it as mannanoligosaccharide and β-glucan triggered promising results. Oral intake of mannanoligosaccharide for two weeks in health BALB/c mice resulted in increased *Lactobacillus* and decreased Enterobacteriaceae, including Gram-negative pathogenic bacteria^24^. The supplementation of turbot larvae fish *Scophthalmus maximus* (Linnaeus, 1758) with a fungal derivative rich in β-glucan altered their microbiota and reduced gene expression of pro-inflammatory cytokines interleukin (IL)-1β and tumor necrosis factor alpha (TNF-α) in total larvae tissue^25^. Also, a yeast-derived β-glucan- and mannan-rich particle called zymosan ameliorated both chronic and relapsing EAE^26^.

Selenium (Se) is a micronutrient essential for physiological processes that due to its antioxidant and immunomodulatory properties has also been considered useful for controlling inflammation^27^. The oral supplementation with Se suppressed immunological and serological features of lupus, as total B cells and autoantibodies, in B6.Sle1b mice model^28^. Concerning microbiota, mice colonized by transplantation with faecal microbiota from Se-enriched supplemented donors showed resistance to dextran sulfate sodium-induced colitis and *Salmonella typhimurium* infection. The protection through microbiota modulation was associated with reduction of IL-1β, IL-6, IL-8 and TNF-α and with increased ZO-1 and claudin-1 expression in the colon, two tight junction proteins involved in the gut barrier function^29^. Considering the properties mentioned above about *S. cerevisiae* and the fact that its effect was not tested in EAE yet, we hypothesized that it could elicit microbiota and immunological modulation capable to control EAE development. We also envisaged that *S. cerevisiae* selenization could improve the supposed beneficial effect of *S. cerevisiae* in EAE. In this scenario, the present research was designed to examine the potential applicability of *S. cerevisiae* and selenium-enriched *S. cerevisiae* (Selemax) in EAE that is the most widely used animal model to investigate new therapies for MS.

## Results

### Clinical EAE development is attenuated by *S. cerevisiae* and its selenized derivative

**Figure 1:**
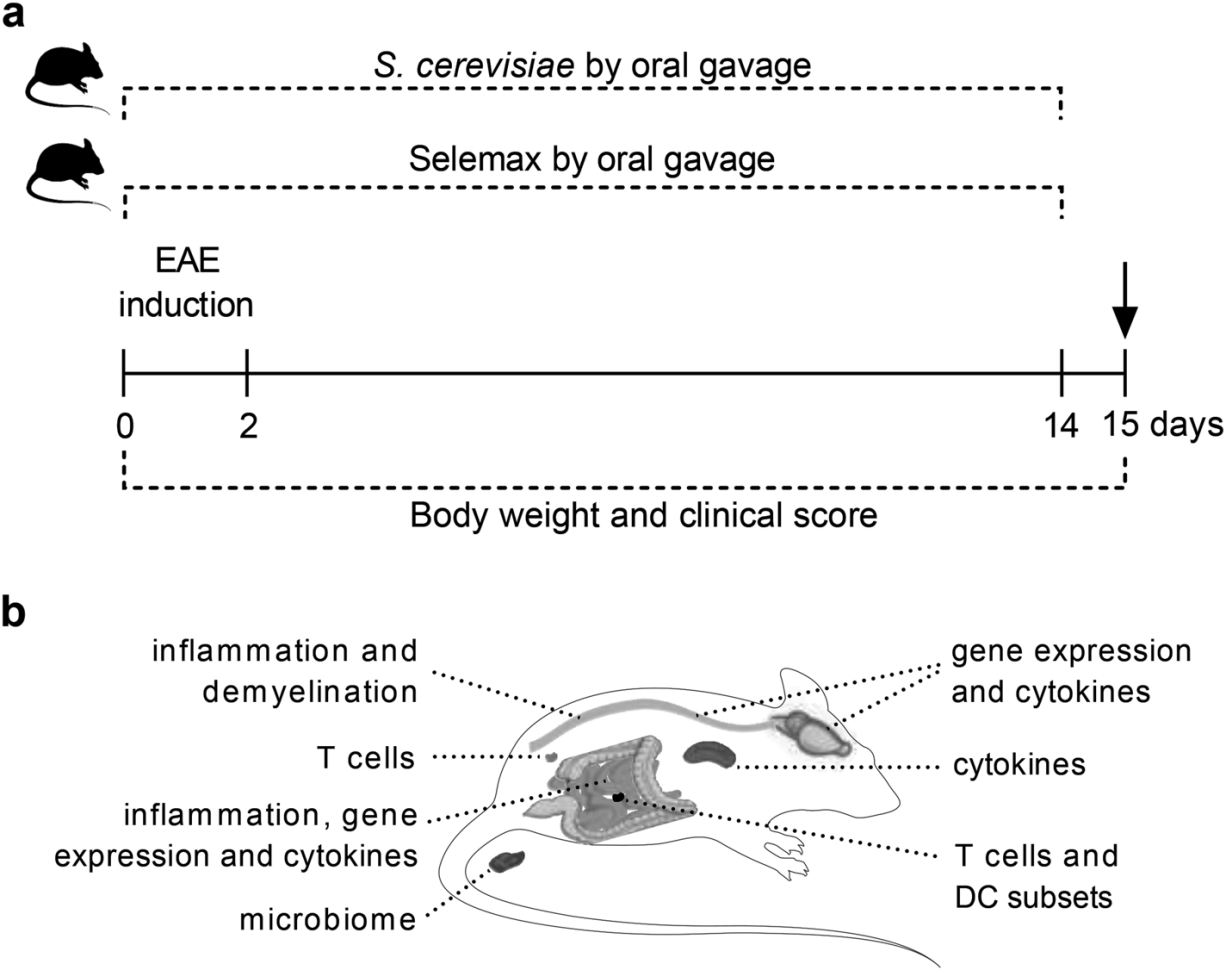
Experimental design. C57BL/6 mice received daily oral doses of *Saccharomyces cerevisiae* or selenium-enriched yeast (Selemax) for 14 days starting on EAE induction (**a**). Body weight and clinical score were assessed daily for 15 days. On the 15th day, which corresponded to the acute disease phase, fresh stools were collected for metagenomic sequencing and the animals were then euthanized (arrow). Samples from gut-brain axis and peripheral lymphoid organs were collected to evaluate immunological parameters (**b**). The mRNA expression and cytokine levels were assessed on CNS (brain plus spinal cord) whereas inflammatory infiltration and demyelination were assessed on lumbar spinal cord. Inflammatory infiltration, mRNA expression and cytokine levels were assessed on small intestine and the proportion of T cells and dendritic cells subsets were evaluated in mesenteric lymph nodes. Peripheral immunological parameters as T cell and cytokine levels were evaluated in inguinal lymph nodes and spleen, respectively. This figure was created using images from Servier Medical Art Commons Attribution 3.0 Unported License (https://smart.servier.com) and image was modified with Adobe Photoshop v.22.0.0.

**Figure 2:**
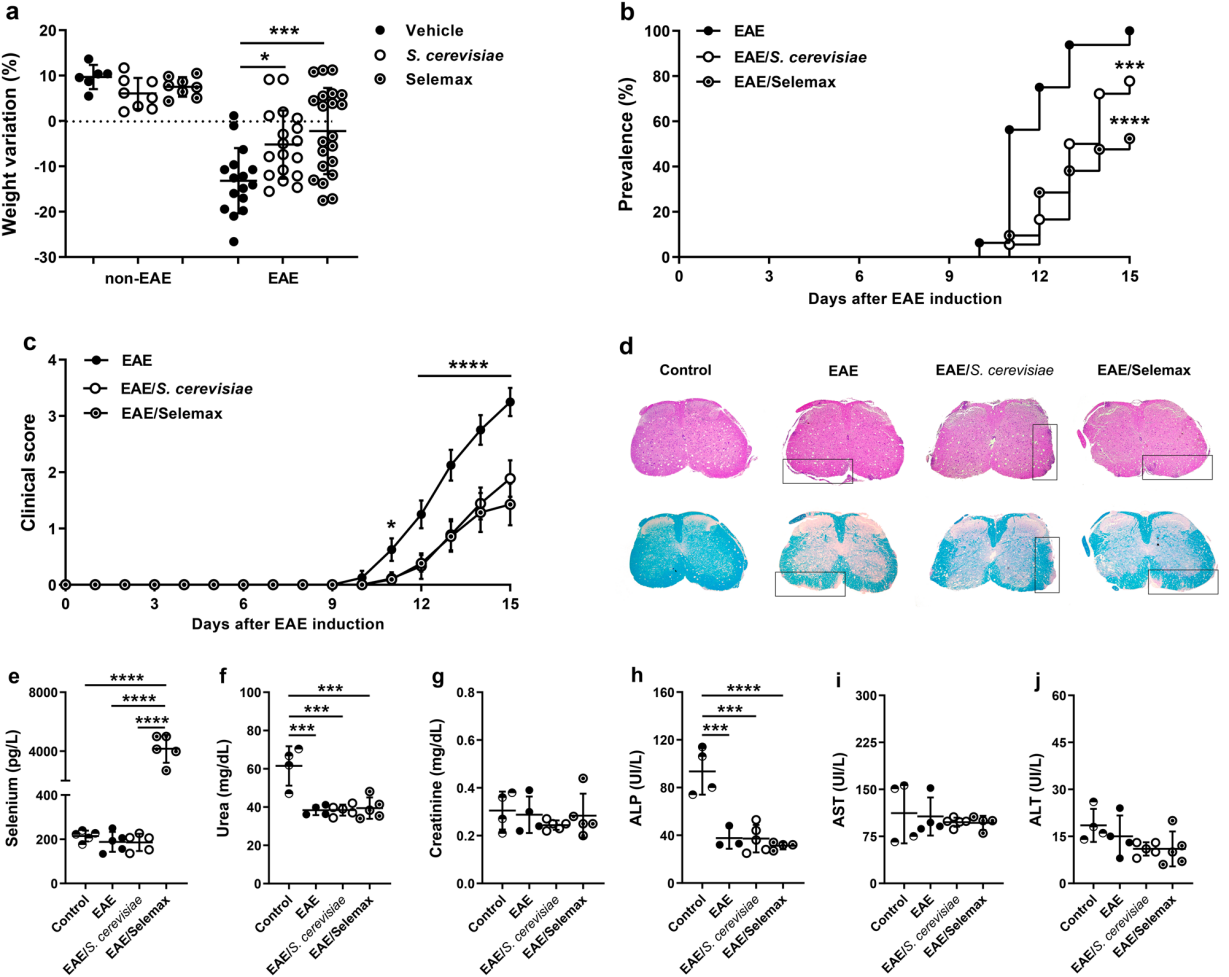
Effect of *S. cerevisiae* and Selemax on EAE development. EAE and non-EAE mice received 14 oral doses of *S. cerevisiae* or Selemax every day. The percentage of weight variation was calculated based on the initial and last animal´s body weight (**a**). Disease prevalence (**b**) and clinical score (**c**) were assessed daily for 15 days. Four independent experiments were combined, n = 6–8 in non-EAE groups and n = 16–21 mice in EAE groups. Inflammatory infiltration and demyelination were evaluated in hematoxylin/eosin (upper) and luxol fast blue (bottom) stained sections, respectively, obtained from control (non-EAE and non-supplemented mice), EAE, EAE/*S. cerevisiae* and EAE/Selemax groups (**d**). Biochemical parameters as total selenium (**e**), urea (**f**), creatinine (**g**), alkaline phosphatase (ALK) (**h**), aspartate aminotransferase (AST) (**i**) and alanine aminotransferase (ALT) (**j**) were analyzed in serum samples. Two independent experiments were combined, n = 4–5 mice/group. Statistical analysis was performed by one-way ANOVA and subsequent Tukey’s test (**a**, **e**) or Holm-Sidak´s test (**f**–**j**), Log-rank (Mantel–Cox) test (**b**) and two-way ANOVA and subsequent Tukey’s test (**c**). All data were expressed as the mean ± SD and statistical differences were represented by *p < 0.05, ***p < 0.001 and ****p < 0.0001. The graphs were created using GraphPad Prism v.8.0.2 and image was modified with Adobe Photoshop v.22.0.0.

To evaluate the effect of supplementation with the yeast in the clinical EAE development, C57BL/6 mice received daily oral doses of *Saccharomyces cerevisiae* or its selenized derivative (Selemax) for 14 days as demonstrated in Fig. 1a. The supplementation by itself did not trigger body weight variation. As expected, EAE mice lost a significant percentage of body weight, which was prevented in both treatments (*p* = 0.0185 and *p* = 0.0006 for *S. cerevisiae* and Selemax, respectively) (Fig. 2a). EAE development, initially assessed by prevalence and clinical score, was also affected by both supplementations. Selemax reduced the disease prevalence by 47.6% (*p* < 0.0001) whereas *S. cerevisiae* reduced by only 22.2% (*p* = 0.0003) in comparison to EAE (Fig. 2b). EAE mice supplemented with *S. cerevisiae* or Selemax developed a significantly milder disease, indicated by lower clinical scores (*p* ≤ 0.0001) (Fig. 2c) and by lower cumulative scores (Table 1) in comparison to non-supplemented EAE mice. These findings were consistent with the histopathological analysis that revealed less inflammation and demyelination in lumbar spinal cord samples from supplemented EAE mice (Fig. 2d). Concerning to the Se concentration, only Selemax supplemented group presented higher serum level of Se in comparison to CTL (*p* < 0.0001), EAE (*p* < 0.0001) and EAE/*S. cerevisiae* (*p* < 0.0001) groups (Fig. 2e). The serum levels of urea and alkaline phosphatase was higher in CTL group in comparison to EAE (*p* = 0.0004 and *p* = 0.0003), EAE/*S. cerevisiae* (*p* = 0.0003 and *p* = 0.0001) and EAE/Selemax (*p* = 0.0004 and *p* < 0.0001) groups (Fig. 2f,h). No difference was observed in relation to creatinine, aspartate aminotransferase and alanine aminotransferase serum levels (Fig. 2g,i,j).

**Table 1:** Cumulative EAE score. Clinical scores were assessed every day during 15 days after EAE induction. The cumulative EAE score was obtained by the sum of daily scores from each mouse in EAE (n = 16), EAE/*S. cerevisiae* (n = 18) and EAE/Selemax (n = 21) groups. Four independent experiments were combined. Two-way ANOVA and subsequent Tukey’s test, *p < 0.05 and **p < 0.01 in comparison to EAE group.

Groups	Days						
	9	10	11	12	13	14	15
EAE	0	2	10	20	34	44	52
EAE/*S. cerevisiae*	0	0	2	6	16*	26*	34*
EAE/Selemax	0	0	2	8	18*	27*	30**

### Downregulation of Th1 in the CNS is more accentuated in Selemax supplemented EAE mice

**Figure 3:**
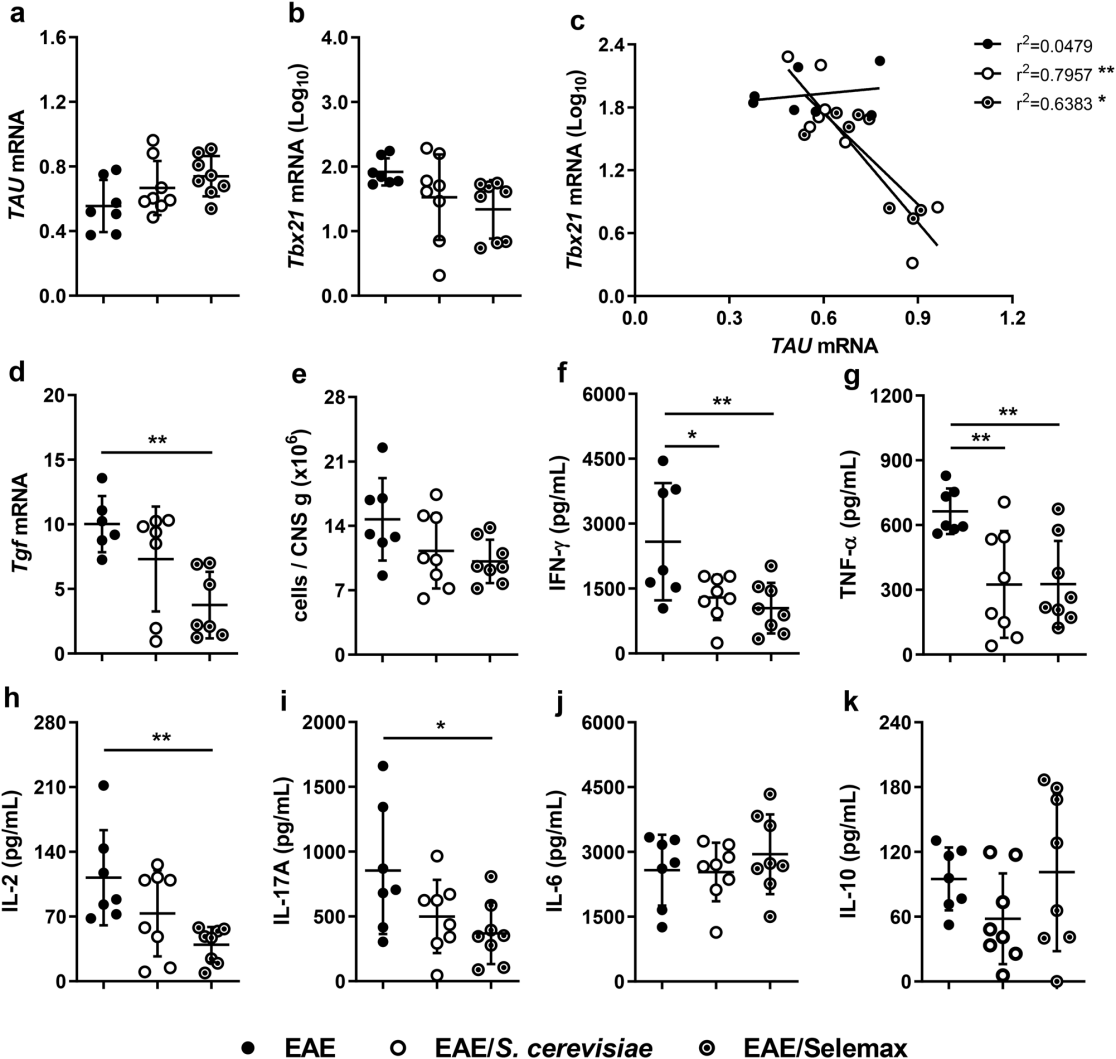
Effect of *S. cerevisiae* and Selemax on CNS inflammatory markers. EAE mice received 14 oral doses of *S. cerevisiae* or Selemax. The mRNA expression of *TAU* (**a**), *Tbx21* (**b**) and *Tgf* (**d**) were performed in lumbar spinal cord samples and the correlation coefficient between *TAU* and *Tbx21* was established (**c**). Total number of leukocytes eluted from the CNS adjusted per gram of tissue (**e**). CNS cell cultures (5 × 10^5^) were stimulated with MOG_35-55_ (50 µg/mL) for 48 h and the production of IFN-γ (**f**), TNF-α (**g**), IL-2 (**h**), IL-17A (**i**), IL-6 (**j**) and IL-10 (**k**) were assessed in the supernatants. Statistical analysis was performed by one-way ANOVA and subsequent Tukey’s test (**a**,**b**,**d**–**j**) and Pearson correlation (**c**). All data were expressed as the mean ± SD and statistical differences were represented by *p < 0.05 and **p < 0.01. Two independent experiments were combined, n = 7–8 mice/group. The graphs were created using GraphPad Prism v.8.0.2 and image was modified with Adobe Photoshop v.22.0.0.

To better characterize the degree of neuroinflammation, we evaluated gene expression in lumbar spinal cord samples and cytokine production by CNS cell cultures, as illustrated in Fig. 1b. An inverse correlation was observed considering the expression of mRNA for *TAU* and *Tbx21* (Th1) in supplemented EAE mice (Fig. 3a-c). mRNA expression for *Rorc* (Th17) and *Foxp3* (regulatory T cells - Treg) was unaffected by any of the supplements, while Selemax significantly decreased *Tgf* (TGF-β) mRNA expression (*p* = 0.0054) (Fig. 3d).

Cells from both experimental groups were adjusted to the same concentration and cultured in the presence of MOG_35-55_ to quantify cytokine production. The number of leukocytes was also significantly lower in the CNS of Selemax supplemented EAE mice (Fig. 3e). Even though IFN-γ and TNF-α were similarly downregulated in EAE/*S. cerevisiae* (*p* = 0.0261 and *p* = 0.0091) and EAE/Selemax (*p* = 0.0079 and *p* = 0.0094) groups (Fig. 3f,g), a significant reduction in IL-2 (*p* = 0.0074) and IL-17A (*p* = 0.0348) levels was only observed in Selemax-treated EAE mice (Fig. 3h,i). No differences were observed in IL-6 and IL-10 levels (Fig. 3j,k).

### Yeast supplementation increases Foxp3^+^ T cells in inguinal lymph nodes

**Figure 4:**
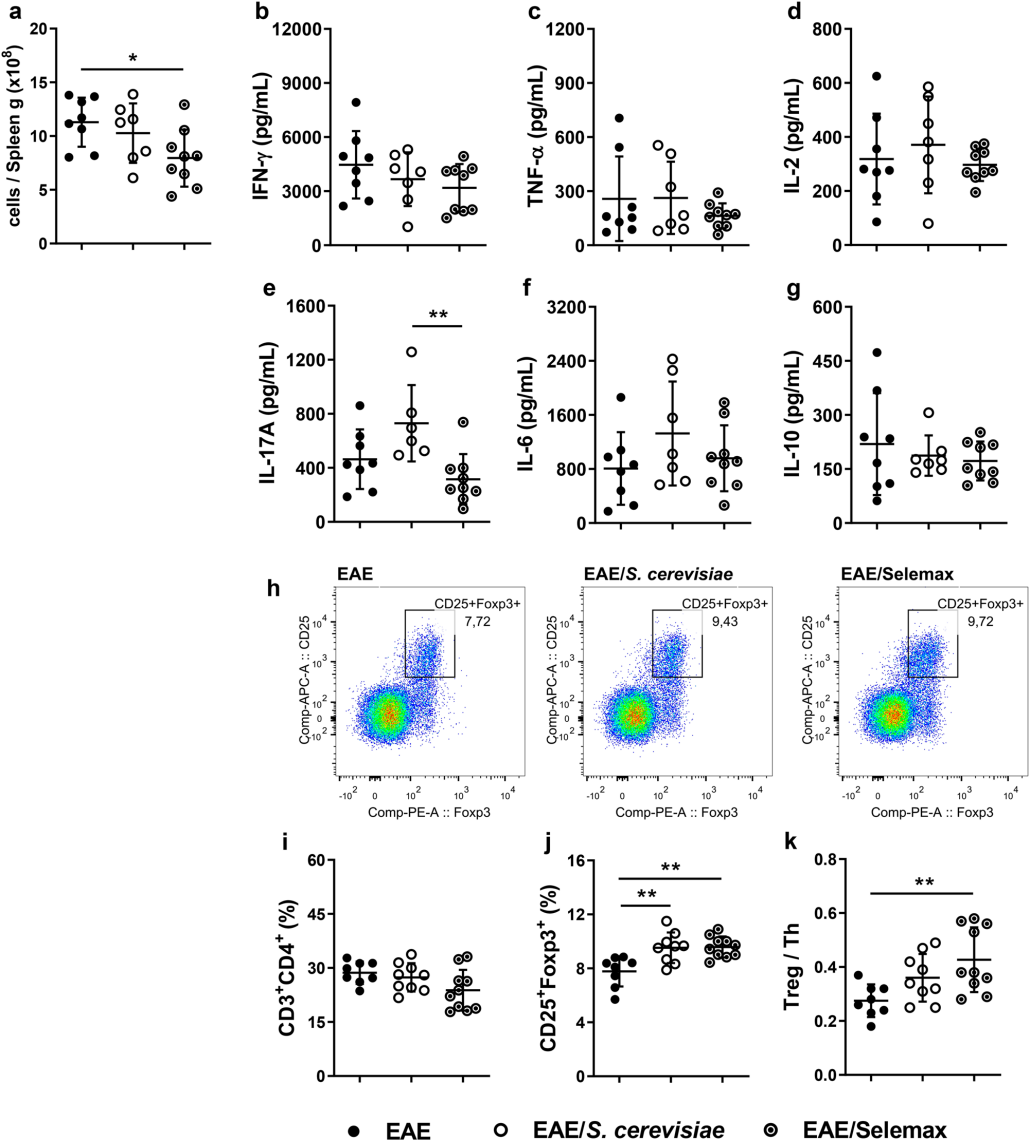
Effect of *S. cerevisiae* and Selemax on cytokine production by spleen and proportion of Tregs in inguinal lymph nodes. EAE mice received 14 oral doses of *S. cerevisiae* or Selemax. Total number of leukocytes from spleen adjusted per gram of tissue (**a**). Spleen cells (5 × 10^6^) were stimulated with MOG_35-55_ (20 µg/mL) for 48 h and the production of IFN-γ (**b**), TNF-α (**c**), IL-2 (**d**), IL-17A (**e**), IL-6 (**f**) and IL-10 (**g**) were assessed in the supernatants. Representative gating strategy to assess regulatory T cells (Treg - CD25^+^Foxp3^+^) in inguinal lymph nodes (**h**). Total cells from inguinal lymph nodes were collected and analyzed by flow cytometry to determine the proportion of T helper cells (Th - CD3^+^CD4^+^) (**i**) and Treg in total Th cells (**j**). The proportion of T subsets was used to calculate the ratio between Treg and Th cells (**k**). Statistical analysis was performed by one-way ANOVA and subsequent Tukey’s test. All data were expressed as the mean ± SD and statistical differences were represented by *p < 0.05 and **p < 0.01. Two independent experiments were combined, n = 7–10 mice/group. The graphs were created using GraphPad Prism v.8.0.2, flow cytometry images were created using FlowJo 10.7.1 and image was modified with Adobe Photoshop v.22.0.0.

To understand the mechanism involved in this protection, we initially characterized the CD4^+^ T cell composition in the spleen and inguinal lymph nodes. The number of total leukocytes was significantly lower in spleen samples from Selemax supplemented EAE mice (*p* = 0.0369) (Fig. 4a). On the other hand, after the stimulation with MOG_35-55,_ splenocytes from EAE, EAE/*S. cerevisiae* and EAE/Selemax groups produced similar amounts of IFN-γ, TNF-α, IL-2, IL-6 and IL-10 (Fig. 4b–d,f,g). The only exception was IL-17A that was produced in lower levels by Selemax supplemented animals than the *S. cerevisiae* supplemented ones (*p* = 0.0064) (Fig. 4e). Even though the proportion of T helper cells (Th - CD3^+^CD4^+^) in inguinal lymph nodes was similar in all experimental groups (Fig. 4i), the proportion of regulatory T cells (Treg - CD25^+^Foxp3^+^) was similarly upregulated by both, *S. cerevisiae* (*p* = 0.0044) and Selemax (*p* = 0.0027) (Fig. 4h,j). Nonetheless, the ratio between Treg and Th cells was higher in the Selemax group (*p* = 0.0069) (Fig. 4k).

### Selenization increases the tolerogenic potential of *S. cerevisiae*

**Figure 5:**
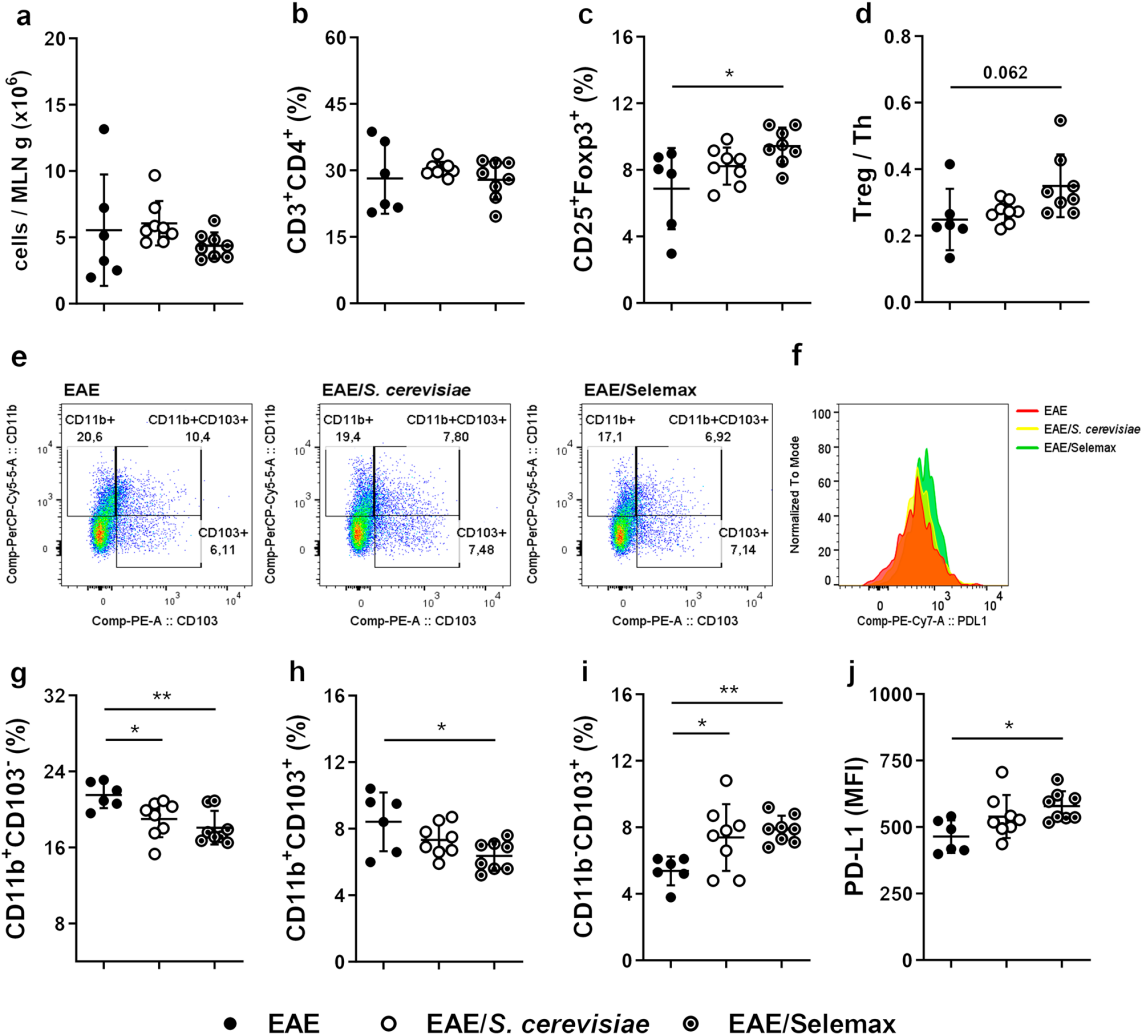
Effect of *S. cerevisiae* and Selemax on the proportion of Tregs and tolerogenic DC in mesenteric lymph nodes. EAE mice received 14 oral doses of *S. cerevisiae* or Selemax. Total number of leukocytes present in mesenteric lymph nodes (MLN) per gram of tissue (**a**). Total cells from MLN were collected and analyzed by flow cytometry to establish the proportion of T helper cells (Th - CD3^+^CD4^+^) (**b**) and regulatory T cells (Treg - CD25^+^Foxp3^+^) (**c**). The proportion of T subsets was used to calculate the ratio between Treg and Th cells (**d**). Representative gating strategy to assess dendritic cell (DC - CD11c^+^MHCII^High^) subsets in inguinal lymph nodes (**e**) and the mean fluorescence intensity (MFI) of PD-L1 in tolerogenic DC (CD11b^-^CD103^+^) subset (**f**). The proportion of CD11b^+^CD103^-^ (**g**), CD11b^+^CD103^+^ (**h**) and CD11b^-^CD103^+^ (**i**) DC subsets in total DC. The MFI of PD-L1 was evaluated in CD103^+^CD11b^-^ DC subset (**j**). Statistical analysis was performed by one-way ANOVA and subsequent Tukey’s test. All data were expressed as the mean ± SD and statistical differences were represented by *p < 0.05 and **p < 0.01. Two independent experiments were combined, n = 6–8 mice/group. The graphs were created using GraphPad Prism v.8.0.2, flow cytometry images were created using FlowJo 10.7.1 and image was modified with Adobe Photoshop v.22.0.0.

Considering that mesenteric lymph nodes (MLN) play a major role as an inducing site of intestinal tolerance^30,31^, our next step was to evaluate the effect of these yeasts in parameters indicative of immunological tolerance. The total leukocyte number and the percentage of CD3^+^CD4^+^ T cells in MLN was similar among groups (Fig. 5a,b). Nonetheless, Selemax supplementation enhanced the proportion of CD4^+^CD25^+^Foxp3^+^ cells (Fig. 5c) and the ratio between Treg and Th cells (Fig. 5d) in comparison to EAE group (*p* = 0.0191 and *p* = 0.0617). In addition, the evaluation of dendritic cell (DC - CD11c^+^MHCII^High^) subsets (Fig. 5e) and the expression of PD-L1 in the subset of tolerogenic DC (CD11b^-^CD103^+^) (Fig. 5f) indicated that selenization can increase the tolerogenic potential of *S. cerevisiae*. Concerning CD11b^+^ DC subsets, the proportion of CD11b^+^CD103^-^ DC (Fig. 5g) was significantly reduced in both EAE/*S. cerevisiae* (*p* = 0.0363) and EAE/Selemax (*p* = 0.0044) groups and CD11b^+^CD103^+^ DC (Fig. 5h) was reduced only in EAE/Selemax (*p* = 0.0141). The percentage of CD11b^-^CD103^+^ tolerogenic DC was higher in both EAE/*S. cerevisiae* (*p* = 0.0379) and EAE/Selemax (*p* = 0.0095) groups in comparison to EAE group (Fig. 5i). Despite this, tolerogenic DC from EAE/Selemax expressed a significantly higher amount of PD-L1 (*p* = 0.0197) in comparison to EAE group (Fig. 5j). The same profile was observed in non-EAE mice (control group). Only Selemax supplementation increased the percentage of CD4^+^CD25^+^Foxp3^+^ cells (*p* = 0.0190) and CD11b^-^CD103^+^ tolerogenic DC (*p* = 0.0502) (Supplementary Fig. 1a and 1b, respectively).

### *S. cerevisiae* and Selemax determine distinct changes in the microbiota

**Figure 6:**
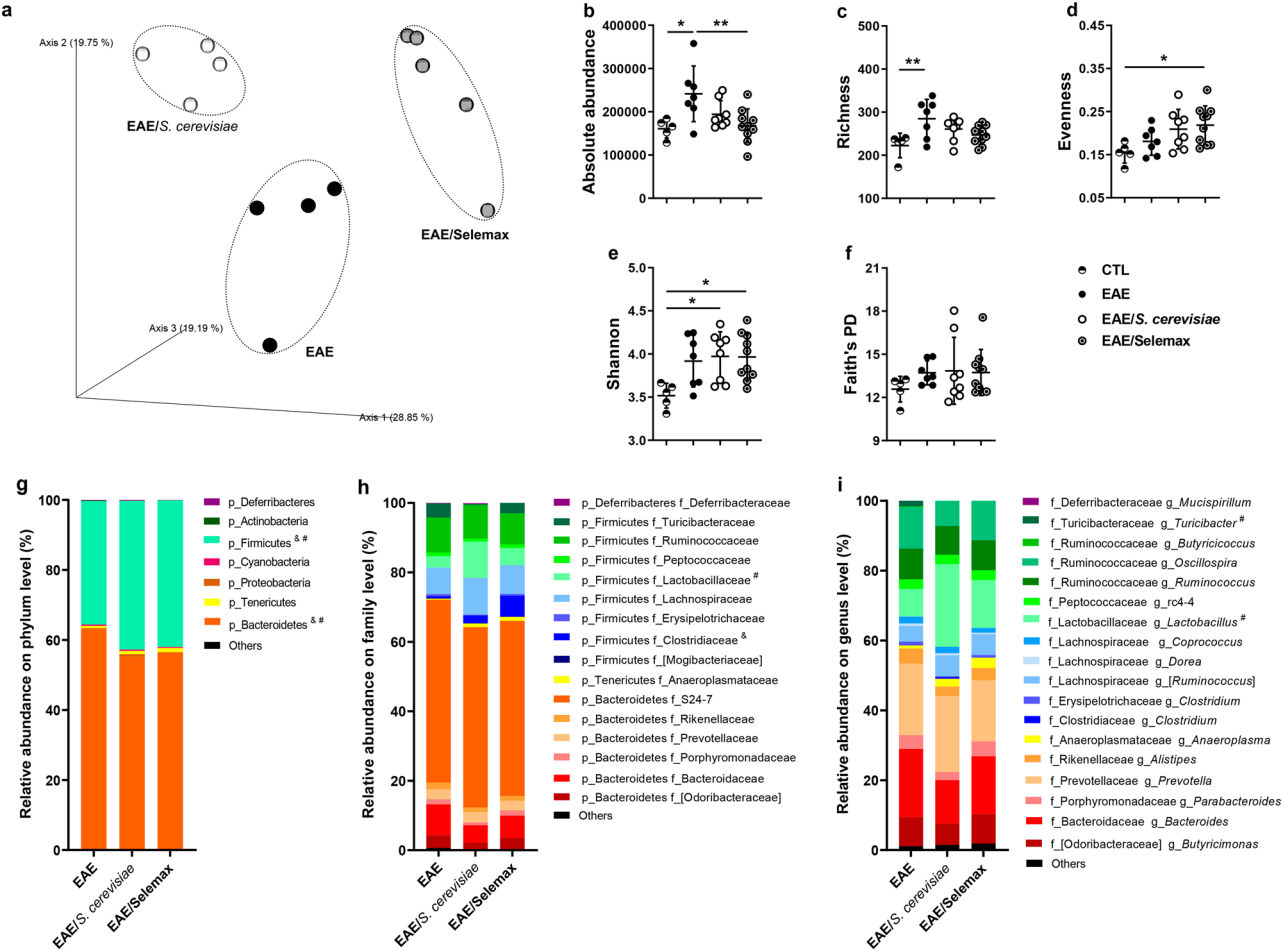
Effect of *S. cerevisiae* and Selemax on microbiota composition. EAE mice received 14 oral doses of *S. cerevisiae* or Selemax. Principal Coordinates Analysis (PCoA) of unweighted UniFrac distances of 16 S rRNA genes (**a**). Absolute abundance (**b**), richness (**c**), evenness (**d**), Shannon diversity index (**e**) and Faith’s phylogenetic index (**f**) of fecal bacterial OTUs. Relative abundance at phylum (**g**), family (**h**) and genus (**i**) level. Statistical analysis was performed by one-way ANOVA and subsequent Tukey’s test (**b**–**f**) and two-way ANOVA and subsequent Tukey’s test (**g**–**i**). All data were expressed as the mean ± SD and statistical differences were represented by *p < 0.05 and **p < 0.01; ^#^* p* < 0.05 between EAE and EAE/*S. cerevisiae*; ^&^p < 0.05 between EAE and EAE/Selemax. One experiment representative of two independent experiments (**a**) and two independent experiments were combined (**b**–**i**), n = 4–9 mice/group. PCoA was created using QIIME2 v.2020.2 (https://view.qiime2.org/), the graphs were created using GraphPad Prism v.8.0.2 and image was modified with Adobe Photoshop v.22.0.0.

Modulation of intestinal immunity by *S. cerevisiae* has been mainly attributed to its ability to change microbiota^32^. Based on this evidence, we used high-throughput sequencing of faecal samples, expressed by operational taxonomic units (OTUs), to investigate the effect of supplementation with *S. cerevisiae* and its selenized derivative on the composition of microbiota. The β-diversity metric, represented by the principal coordinate analysis (PCoA), indicated that microbiota was different among the three groups (Fig. 6a). Permutational multivariate analysis of variance (PERMANOVA) revealed that the bacterial community structure in EAE group was different from EAE/*S. cerevisiae* (*p* = 0.029) or EAE/Selemax (*p* = 0.011) groups and between the two supplemented groups (*p* = 0.014). Regarding α-diversity metrics, were evaluated the absolute abundance, richness, evenness, Shannon–Wiener diversity index and Faith’s phylogenetic diversity index. EAE group had increased absolute abundance (absolute number of bacteria in the sample) and richness (the number of OTUs) in comparison to CTL group (*p* = 0.0167 and *p* = 0.0098) (Fig. 6b,c). At the family level these differences were mainly related to the detection of OTUs in Christensenellaceae, Coriobacteriaceae, Erysipelotrichaceae, Lachnospiraceae, Ruminococcaceae and S24-7 in EAE group but not in CTL group; whereas at genus level, the same was observed concerning *Adlercreutzia*, *Anaerostipes*, *Coprococcus*, *Dorea*, *Oscillospira* and *Ruminococcus* genera. The supplementation with Selemax decreased the absolute abundance in EAE mice (*p* = 0.0084) (Fig. 6b) but did not alter the richness. Additionally, the supplementation with Selemax increased evenness (*p* = 0.0309) and both supplementations, *S. cerevisiae* and Selemax, increased Shannon–Wiener diversity index (*p* = 0.0279 and *p* = 0.0234, respectively) in comparison to CTL group (Fig. 6d,e). The phylogenetic diversity (Faith’s phylogenetic diversity index) did not differ between EAE and supplemented groups (Fig. 6f).

Lastly, we analyzed the relative abundance of taxonomic groups in the supplemented EAE-mice. At the phylum level (Fig. 6g), Bacteroidetes relative abundance in EAE mice was higher than EAE mice supplemented with *S. cerevisiae* (*p* = 0.0044) or Selemax (*p* = 0.0135) whereas the Firmicutes phylum was lower in EAE group in comparison to EAE/*S. cerevisiae* (*p* = 0.0110) or EAE/Selemax (*p* = 0.0163) groups. At the family level, we observed an increase in the relative abundance of Lactobacillaceae in EAE/*S. cerevisiae* (*p* = 0.0017) and Clostridiaceae in EAE/Selemax group (*p* = 0.0176) in comparison to EAE group (Fig. 6h). When differences were evaluated at the genus level, only *S. cerevisiae* supplementation triggered a significant microbiota modulation. The genus *Turicibacter* was reduced (*p* = 0.0024), whereas the *Lactobacillus* one was increased (*p* < 0.0001) in EAE/*S. cerevisiae* in comparison to EAE group (Fig. 6i).

### Selemax controls intestinal inflammation better than *S. cerevisiae*

**Figure 7:**
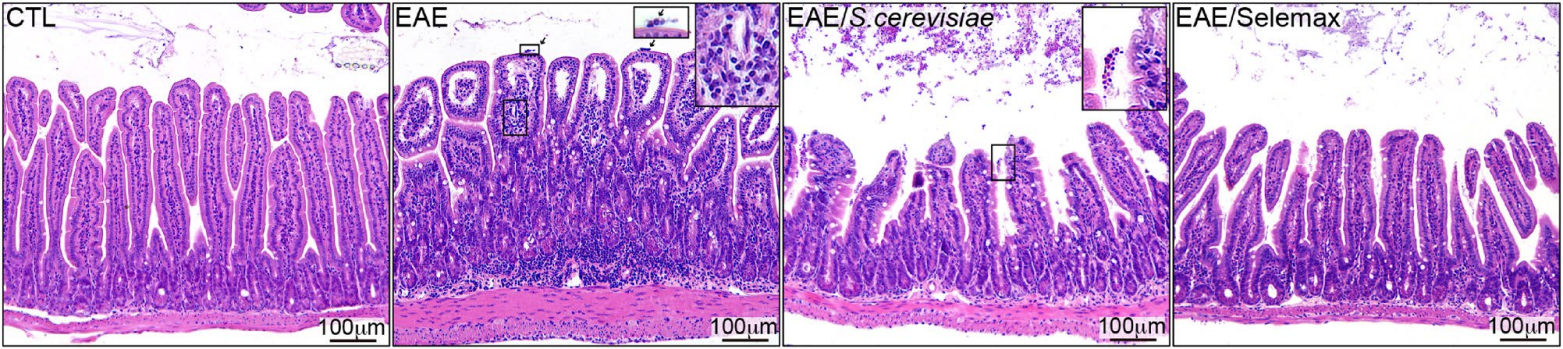
Effect of *S. cerevisiae* and Selemax on small intestine inflammation. EAE mice received 14 oral doses of *S. cerevisiae* or Selemax. The inflammatory infiltrate of the lamina propria of the small intestine was evaluated using hematoxylin/eosin stained sections of control – CTL (non-EAE and non-supplemented mice), EAE, EAE/*S. cerevisiae* and EAE/Selemax groups. One independent experiment, n = 4–5 mice/group. Image was created with Adobe Photoshop v.22.0.0.

Considering that both yeasts increased the diversity of microbiota and altered the relative abundance of some bacterial groups, we evaluated whether microbiota modulation was related to different degrees of intestinal inflammation. When compared to the healthy control group, the villus of EAE animals were enlarged, presented reduction in length and displayed a significant increase in mononuclear and polymorphonuclear cells, suggesting the presence of neutrophils infiltrating the lamina propria as well as the submucosa. In addition, an influx of such polymorphonuclear cells was detected in the gut lumen of EAE mice (arrows in the second panel), which is indicative of severe inflammation. *S. cerevisiae* and Selemax-supplemented mice presented a significant reduction in the cell infiltrate in the lamina propria however, the intestinal structure was still altered in comparison to non-EAE group (Fig. 7). Selemax supplementation was slightly more effective in attenuating gut inflammation. Notably, we also detected the presence of yeasts in both treated groups (as indicated in the upright insert in the third panel) suggesting, therefore, their gut accumulation after treatment.

**Figure 8:**
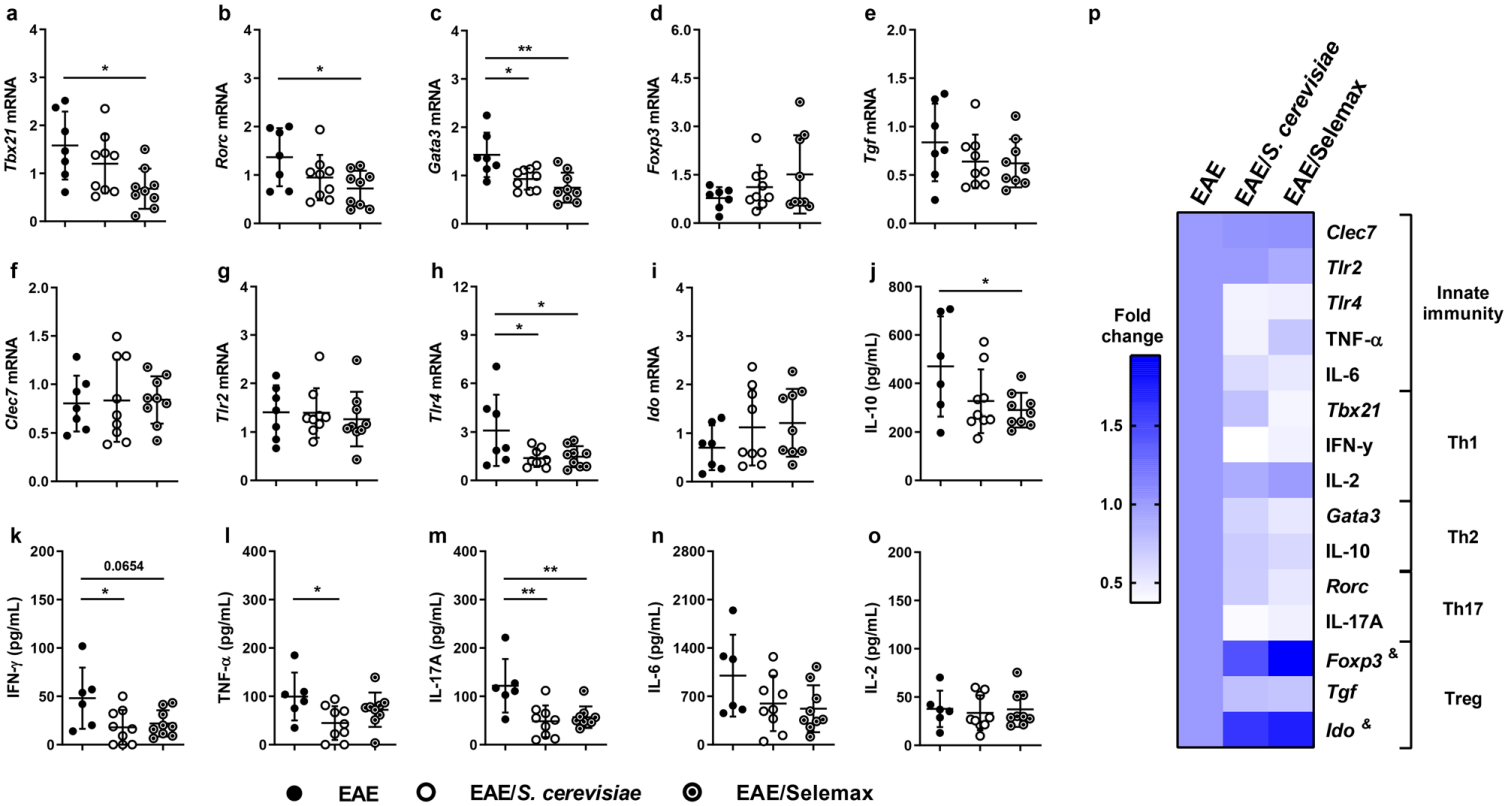
Effect of *S. cerevisiae* and Selemax on intestinal mucosal inflammatory markers. EAE mice received 14 oral doses of *S. cerevisiae* or Selemax. The mRNA expression of *Tbx21* (**a**), *Rorc* (**b**), *Gata3* (**c**), *Foxp3* (**d**), *Tgf* (**e**), *Clec7* (**f**), *Tlr2* (**g**), *Tlr4* (**h**) and *Ido* (**i**) were performed in small intestine samples. Gut explants of small intestine were cultured for 6 h and the production of IL-10 (**j**), IFN-γ (**k**), TNF-α (**l**), IL-17A (**m**), IL-6 (**n**) and IL-2 (**o**) were assessed in the supernatants. The gene expression and cytokine levels were used to calculate the fold change in relation to EAE group (**p**). Statistical analysis was performed by one-way ANOVA and subsequent Tukey’s test (**a**–**o**) and two-way ANOVA and subsequent Tukey’s test (**p**). All data were expressed as the mean ± SD and statistical differences were represented by *p < 0.05 and **p < 0.01; ^&^* p* < 0.05 between EAE and EAE/Selemax. Two independent experiments were combined, n = 7–8 mice/group. The graphs were created using GraphPad Prism v.8.0.2 and image was modified with Adobe Photoshop v.22.0.0.

Gut-homing of myelin-specific Th17 has been described as a pivotal step for the development of EAE^33^. Assays involving gene expression in intestinal samples and cytokine production by gut explant cultures were then performed to evaluate if supplementation was affecting the local level of T cell subsets, including Th17 and Treg, and of immunoregulatory molecules. A significant reduction was observed in the expression of *Tbx21* (*p* = 0.0151) (Fig. 8a), *Rorc* (*p* = 0.0338) (Fig. 8b) and *Gata3* (Th2) (*p* = 0.0015) (Fig. 8c) mRNA levels in Selemax supplemented EAE-mice compared to non-supplemented EAE-animals. No differences were observed among the three experimental groups concerning the expression of genes related to Treg cells as *Foxp3* (Fig. 8d), *Tgf* (Fig. 8e) and *Ido* (indoleamine 2,3-dioxygenase) (Fig. 8i). The expression of pattern recognition receptors (PRRs) involved in the interaction with *S. cerevisiae* was also evaluated. Both products significantly reduced the expression of *Tlr4* (*p* = 0.0451) (Fig. 8h) but not any other PRRs measured, like *Clec7* (Fig. 8f) and *Tlr2* (Fig. 8g).

Corroborating with the findings of gene expression, gut explant immersion cultures from EAE mice supplemented with Selemax showed reduced production of IFN-γ (*p* = 0.0654) (Fig. 8k), IL-17A (*p* = 0.0074) (Fig. 8m) and IL-10 (*p* = 0.0513) (Fig. 8j) when compared to the untreated EAE group. Similarly, the production of IFN-γ (*p* = 0.0309), TNF-α (*p* = 0.0369) and IL-17A (*p* = 0.0027) were significantly reduced in *S. cerevisiae* supplemented group (Fig. 8k–m), compared to the EAE group. No differences were observed in IL-6 (Fig. 8n) and IL-2 (Fig. 8o) production in both supplemented groups in comparison to EAE animals. Gene expression and cytokine production were used to calculate the fold change of data from supplemented groups in relation to the EAE group (Fig. 8p). This overview of fold change clearly revealed that Selemax supplementation improved the benefits of *S. cerevisiae* in the context of EAE. As can be observed, the selenized yeast decreased most inflammatory parameters and simultaneously increased tolerogenic indicators in the intestinal environment, as *Foxp3* (*p* = 0.0010) and *Ido* (*p* = 0.0161) (Fig. 8p).

## Discussion

In the current study, we evaluated the therapeutic potential of *Saccharomyces cerevisiae* and its selenized derivative named Selemax to control EAE development. Both yeast preparations were clearly able to control clinical disease evolution. To the best of our knowledge, this is the first report supporting the usage of *S. cerevisiae* and Selemax to control experimental MS. This seems a relevant contribution because yeast-rich diets or yeast supplementation have been used for a long time worldwide, in a series of settings, without side effect records. *S. cerevisiae* is one of the most well-known yeast being predominantly employed for food fermentation and used for centuries in bread-making and production of alcoholic beverages such as wines and beers^34^. Besides the use of wild type strains, engineered *S. cerevisiae* strains are being largely employed as commercially profitable cell systems for application in bioprocesses aiming the synthesis of biopharmaceuticals and therapeutic proteins^35,36^. Another application of *S. cerevisiae* is its enrichment with essential elements for the organism's homeostasis, such as iron^37^ and selenium (Se)^38,39^. The enrichment of *S. cerevisiae* yeast with Se has been previously reported and increased its immunomodulatory potential. The oral administration of *S. cerevisiae* protected mice against mucositis and reduced the inflammatory response in the intestinal tissue and this effect was potentiated when it was enriched with Se^40^. Se has antioxidant and anti-inflammatory properties which are particularly relevant in CNS diseases^41^. Only a few reports have addressed the relevance of Se levels in MS patients. Significantly lower serum levels were described in one study^42^ whereas no alterations were found in two other investigations, in comparison to healthy controls^43,44^. In the present work, no differences were observed concerning serum Se concentration between non-EAE and EAE-mice. As expected, only Selemax supplemented group presented higher serum level of Se. Of note, these high Se levels were not associated with an evident renal or hepatic toxicity.

We hypothesized that the Se-enriched yeast could be a safe approach to achieve modulation of both, microbiota and autoimmune response in the context of EAE. Our initial findings already suggested that selenization was improving *S. cerevisiae* ability to control EAE development; this was indicated by the lower paralysis prevalence in this experimental model. Clinical protection in both supplemented groups was associated with less inflammation and demyelination in the CNS. Besides, a more detailed analysis of the cells eluted from this tissue confirmed the lower inflammatory process in the Selemax supplemented EAE-mice. They presented a reduced number of leukocytes in the CNS, including Th1 cells, and produced less encephalitogenic cytokines. In addition, the level of *TAU* mRNA was statistically higher in the EAE/Selemax group. Tau belongs to the microtubule-associated proteins family, essential for microtubule assembly in neurons. Usually found in axons, the protein can also be translocated to the cell body and dendrites and the abnormal deposition of modified tau in the neurons is a common aspect of many neurodegenerative diseases called “tauopathies”^45^. The role of this protein has also been investigated in EAE/MS. Mice devoid of tau protein presented higher susceptibility to neuronal damage, suggesting that tau integrity is relevant to avoid axonal damage in EAE mice^46^. In MS patients, the lower levels of tau in the cerebrospinal fluid have been correlated with parenchymal brain loss^47^ and may denote axonal degeneration^48^. In addition, we observed a lower expression of *tgf* in CNS samples from Selemax supplemented EAE-mice. It has been described that early production of TGF-β1 by glial cells creates a dangerous environment for the initiation of autoimmune inflammation whereas pharmacological inhibition of TGF-β signaling ameliorated the disease signs and reduced pathogenic T cells and IL-6 expression in the CNS^49^. The reduction of neuroinflammation and increased *TAU* expression in CNS observed in EAE-mice supplemented with Selemax support the enhanced protective effect of Se-enriched yeast in relation to non-enriched *S. cerevisiae* yeast and reinforce the relevance of our findings.

The ensuing experiments were done to investigate if these distinct levels of protection were due to differential immunomodulatory ability on the spleen or the regional lymph nodes. Even though a few differences were observed between *S. cerevisiae* and Selemax supplemented mice concerning the number of spleen cells and proportion of Treg (CD25^+^Foxp3^+^), they did not seem relevant enough to explain the most pronounced protective effect of selenized yeast. However, the data obtained from the MLN revealed a higher percentage of Treg and tolerogenic DC (CD11b^-^CD103^+^) in Selemax than in *S. cerevisiae-*supplemented mice. These findings are in consonance with literature data that supports the contribution of these cells to control inflammation in EAE and MS^20,50,51^. In addition, these tolerogenic DC expressed higher levels of PD-L1. The contribution of PD-L1 expression in DC as a mechanism to limit the initial activation of myelin-reactive CD4^+^ T cells was already reported in EAE^52^. Also, among four distinct DC subsets expressing CD103 and/or PD-L1 in MLN, only CD11b^-^CD103^+^PD-L1^High^ DC subset was a higher inducer of Foxp3^+^ T cells^53^. These findings may sugest that selenization is acting, at least partially, by increasing the DC/Treg pathway. Findings of previous literature reports support this viewpoint. In an iodine-induced autoimmune thyroiditis model, the percentage of Treg cells and the expression of *Foxp3* mRNA were increased by treatment with sodium selenite in drinking water^54^. As reviewed by Nettleford and Prabhu^55^, Se acts on intestinal immunity through upregulation of the nuclear hormone receptor peroxisome proliferator-activated receptor gamma leading to differentiation of Treg and inhibition of Th1 and Th17. Se also inhibits NF-κB, decreasing, therefore, the production of pro-inflammatory cytokines that are required for T helper cell differentiation.

To better understand the mechanism by which these two supplements were being protective and possibly help to elucidate the greater effectiveness of Selemax, we analyzed the faecal microbiota. The results clearly indicated that both supplements were able to increase microbiota diversity in EAE-mice, but only Selemax was able to reduce the enhanced absolute abundance associated with the disease. A diversified microbiota is generally considered beneficial to the health^56,57^; conversely, the decline in microbiota diversity has been linked to the rise of modern diseases as obesity, asthma, food allergies, diabetes, inflammatory bowel disease, and cognitive disorders^58^. A low-diversity microbiota contributes to decreasing microbiota resilience and stability and changes the growth of some microbes relative to others^59^. Recently, a number of studies showing that MS patients and EAE mice exhibit gut dysbiosis disclosed the gut microbiome as a relevant player in the development of this disease^60–64^. In the present work, EAE development was associated with an increased absolute abundance and richness and the detection of some bacteria not observed in non-EAE mice. EAE mice exhibited dysbiosis associated, at least partially, to the detection of an increased abundance of *Dorea* and *Ruminococcus* genera, similarly to what is observed in patients with MS^62,63^. Considering that the disease causes dysbiosis related to bacterial abundance, we evaluated if yeast supplementation altered the relative abundance of bacterial community at different taxonomic levels. At the phylum level, both supplements increased Firmicutes and decreased Bacteroidetes phyla. Considering that the Bacteroidetes phylum has been related to an inflammatory profile^65^, the reduction of this taxon may have contributed to protection. A higher abundance of Lactobacillaceae and Clostridiaceae families, both from the Firmicutes phylum, was detected in faecal samples of *S. cerevisiae* and Selemax supplemented EAE groups, respectively. Considering that members of Lactobacillaceae and Clostridiaceae family were significantly decreased in MS patients^63^, their increase in supplemented EAE groups could be related to protection. Results observed in experimental diseases, including EAE, support this possibility. For instance, the oral administration of heat-killed *Candida kefyr* to EAE mice resulted in disease protection associated with higher Lactobacillales abundance and increased Treg proportion^20^. The direct role of *Lactobacillus* in inflammation has been linked to the differentiation of Treg cells in MLN of mice fed with *L. casei*^66^ and related to autoimmunity suppression after remodelling microbiota with *L. reuteri* administration in Treg-deficient scurfy mice^67^ and in EAE mice^21^. Similarly, the oral administration of 17 Clostridia strains to adult mice provided bacterial antigens and a TGF-β-rich environment that helped Treg cells expansion and differentiation and attenuated other experimental diseases, as colitis and allergic diarrhea^68^. The induction of Treg by Clostridia has been attributed to signals delivered by CD103^+^ DC to drive naïve CD4^+^ T cells to differentiate into Treg cells^69^. At genus level, only supplementation with *S. cerevisiae* increased *Lactobacillus* and reduced *Turicibacter*. Despite this, both supplements significantly increased Firmicutes phylum and families of this phylum, and, as mentioned above, members of these families have been related to the regulation of inflammation.

Having in mind that MLN are a pivotal site in intestinal immunity^70^, that DC traffic from the intestine to these lymph nodes^71^, and that Th1 cells migrate to the gut contributing to the inflammatory environment that favours Th17 differentiation during EAE development^72^, we evaluated the effect of supplementation on the intestinal inflammatory condition. The histopathological analysis revealed that the intestinal inflammation degree was lower in supplemented animals being this effect more evident in the EAE/Selemax group. This finding was reinforced by other assays performed with gut explants that showed lower levels of proinflammatory gene expression and of cytokine production in supplemented EAE mice. Some of these parameters, as IL-6, IL-10, IL-17A, *Tbx21*, *Rorc*, and *Gata3* were more downmodulated in EAE/Selemax mice than in EAE/*S. cerevisiae* ones. The decreased expression of toll-like receptor (*TLR)-4* in both supplemented groups seems meaningful. Considering that the expression of *Tlr4* is low at the small intestine in a steady-state condition^73^, we believe that most of its elevated expression in EAE mice could be due to the presence of inflammatory cells, mainly CD4^+^ T lymphocytes and γδT cells. This possibility is supported by data that indicates a T cell homing stage during EAE development^33,72^. In the EAE model, the absence of TLR4 solely in CD4^+^ T cells significantly decreased disease signs; the authors also found that Tlr4^-/-^ γδT cells presented defective IFN-γ and IL-17 production^74^. In addition to the reduction of innate and adaptive components of the immune system, Selemax also increased the fold change of *Foxp3* and *Ido* mRNA expression in the small intestine. This seems interesting because it has been shown that encephalitogenic cells migrating into the gut-associated lymphoid tissue can be restrained there, expelled into gut lumen and even transformed into Treg which are effective against CNS autoimmunity^75,76^. CNS and gut are connected via the gut–brain axis with bidirectional interactions between the CNS and the gastrointestinal tract, including microbial community^77,78^. These bidirectional interactions enable the brain to influence gastrointestinal functions as well as the immune functions through the cross-talk amongst the gut, the nervous system, and the immune system^79^.

In summary, *S. cerevisiae* increased Lactobacilaceae whereas selenium-enriched yeast (Selemax) increased Clostridiaceae and both supplements increased Treg proportion in MLN and controlled EAE development. However, the prevalence of the disease was lower in Selemax supplemented group and only these mice showed a higher PD-L1 expression on CD103^+^ DC in MLN and a significant reduction of IL-17A in the CNS and IL-17A and *Rorc* in the small intestine. In this context, Selemax supplementation seems to be a formulation worthwhile to be considered for MS therapy once it is endowed with modulatory properties towards the microbiota and the immune system. It is important to mention, however, that Se status can be affected by several factors, including genetic variation. This polymorphism affects mainly the selenoproteins involved in its metabolism^80^. These genetic differences must be checked and considered in any preventive or therapeutic measure involving supplementation with Selemax or other organic Se sources.

Altogether our results show that selenization of *S. cerevisiae* improved its ability to control EAE development. These data also suggest that a significant part of this protective effect seems to occur at the intestinal level, including the generation of a tolerogenic scenario at the MLN and small intestine and modifications of the gut microbiota. In this context, this selenized yeast is a strong candidate to be trialed, in the future, as a CAM in MS patients.

## Methods

### Experimental design

C57BL/6 mice were allocated into six experimental groups: control (CTL)—healthy animals (n = 6); *S. cerevisiae*—healthy animals daily supplemented with *Saccharomyces cerevisiae* (n = 8); Selemax—healthy animals daily supplemented with selenized *S. cerevisiae* (n = 8); experimental autoimmune encephalomyelitis (EAE)—animals immunized with myelin oligodendrocyte glycoprotein peptide (MOG_35-55_) to develop encephalomyelitis (n = 16); EAE/ *S. cerevisiae*—immunized with MOG and daily supplemented with *S. cerevisiae* (n = 18), and EAE/Selemax—immunized with MOG and daily supplemented with selenized *S. cerevisiae* (n = 21). Four independent experiments were combined. Supplementation was orally delivered by gavage for 14 days, once a day. CTL and EAE groups received the diluent in the same conditions. Body weight and clinical score were evaluated daily for 15 days. On the 15th day, that corresponded to the acute phase of EAE, fresh faecal samples were collected for metagenomic sequencing. The animals were then euthanized with an overdose of ketamine–xylazine and blood samples were collected through cardiac puncture. Mice were then perfused intracardially with sterile Phosphate Buffered Saline (PBS) before samples collection. Lumbar spinal cord was used for histopathological analysis and real time polymerase chain reaction (PCR) assays and brain plus cervical and thoracic spinal cord eluted cells were cultured to assess cytokine production. Spleen cells were cultured to analyze peripheral cytokine production and inguinal lymph node cells were used to evaluate T cell subsets by flow cytometry. T and dendritic cell subsets present in mesenteric lymph nodes were also analyzed by flow cytometry. Pieces (4–5 mm) of small intestine (jejunum and ileum) were freshly used for cultures to assess cytokine production, fixed for histopathological analysis or frozen for further PCR assays. The experimental design is summarized in Fig. 1 and in Supplementary Table 1.

### Animals

Six-week-old female C57BL/6 mice were purchased from a specific pathogen-free facility at the University of São Paulo (USP) (Ribeirão Preto, SP, Brazil). The mice were housed (4 mice/cage) in a ventilated rack system (Alesco, Monte Mor, SP, Brazil) in the animal facility of the Department of Chemical and Biological Sciences of the São Paulo State University (UNESP) (Botucatu, SP, Brazil). Animals received sterilized maintenance diet (AIN-93 M)^81^ and water ad libitum. All protocols involving mice followed the guidelines of National Council to Control Animal Experimentation (CONCEA, Brazil) and were approved by the local Committee on Ethics in the Use of Animals (CEUA – protocol number 1033/2017), (Institute of Biosciences, Botucatu, SP, Brazil).

### EAE induction and evaluation

Eight-week-old mice were subcutaneously immunized, into the lower back, with 100 μg of myelin oligodendrocyte glycoprotein peptide (MOG_35–55_ – MEVGWYRSPFSRVVHLYRNGK) (Genemed Synthesis Inc., San Antonio, TX, USA) emulsified in 25 μL of complete Freund’s adjuvant (Sigma-Aldrich, St. Louis, MO, USA) containing 2 mg/mL of *Mycobacterium tuberculosis* (Difco, Detroit, MI, USA). Immediately and 48 h after immunization, the animals were intraperitoneally injected with 200 ng of *Bordetella pertussis* toxin (Sigma-Aldrich). Clinical scores were assessed daily for 15 days according to the following criteria^82,83^: no signs (0); limp tail (1); hind legs weakness (2); partially paralyzed hind legs (3); complete hind legs paralysis (4); and complete paralysis or death (5). Body weight loss was calculated considering the difference between the initial weight (at the time of EAE induction—day 0) and the one at the acute disease phase (day 15).

### Supplementation with *S. cerevisiae* and Selemax

*Saccharomyces cerevisiae* (YSC 11,111) and Selemax, a selenium-enriched *S. cerevisiae*-YSC111, were provided by Biorigin (Zilor, Lençóis Paulista, SP, Brazil). Selemax production was performed according to Oraby et al.^39^. Both supplements were diluted in sterile water and administered once a day to the animals by gavage for 14 days. The daily dose of both supplements was 21.34 mg. In the case of Selemax this dose contained 50 µg of Se that, according to Vieira et al.^84^, was therapeutically effective in experimental arthritis. The composition of both supplements is described in Table 2.

**Table 2:** Supplements composition.

	*S. cerevisiae*	Selemax
Glucans	12.5%	12.3%
Mannan	14.8%	15.1%
Selenium	–	2343 mg/kg*

*2343 mg of selenium per kg of *S. cerevisiae*.

### Histopathology

Lumbar spinal cord and small intestine (jejunum and ileum) samples were aseptically removed and fixed in 10% neutral buffered formalin. Tissues were dehydrated in graded ethanol, diaphanized in xylol and embedded in Paraplast Plus (McCormick, St. Louis, MO, USA). Paraffin slides with 4 µm were stained with hematoxylin and eosin (H&E) or stained with Luxol Fast Blue (LFB) to assess the inflammatory process and demyelination, respectively. Three photomicrographs were obtained from each animal with Olympus BX60 microscope.

### Serum dosages

Total selenium in blood serum was carried out using a quadrupole inductively coupled plasma mass spectrometer (ICP-QMS, Agilent, Santa Clara, CA, USA) and^77^ Se as the isotope of choice. Concentrations of urea, creatinine, alkaline phosphatase, aspartate aminotransferase and alanine aminotransferase serum levels were quantified with Bioclin commercial kits (Quibasa Química Básica Ltda., Belo Horizonte, MG, Brazil) and results were measured by Cobas Mira Plus Chemistry Analyzer (Roche Diagnostics, Basel, Switzerland).

### Splenocyte culture

Spleens were dissociated with sterile pestles and the red blood cells lysed with 1 mL of ammonium chloride buffer at room temperature for 5 min. The remaining cells were washed with RPMI 1640 (Sigma-Aldrich) medium and then adjusted to 5 × 10^6^ cells/mL in RPMI 1640 medium supplemented with 10% heat-inactivated fetal calf serum (Gibco BRL, Grand Island, NY, United States), 2 mM of l-glutamine (Sigma-Aldrich) and 0.1% antibiotic/antimycotic (Sigma-Aldrich). Spleen cell suspensions were distributed in 48-well plate, stimulated with MOG_35-55_ (20 µg/mL) and incubated at 37 °C in a humidified incubator containing 5% CO_2_. After 48 h incubation, supernatants were stored at − 20 °C for further cytokine quantification.

### CNS cell cultures

Brain and spinal cord were collected and digested with 2.5 mg/mL of collagenase D (Roche Applied Science, Indianapolis, IN, USA) and 100 μg/mL of DNAse (Sigma-Aldrich) at 37 °C for 45 min. The digested tissue homogenate was gently pressed through a 70-µm cell strainer and washed with 5 mL of Hank's Balanced Salt Solution (HBSS) at 18 °C, 450 × g for 7 min (centrifuge 5810R, Eppendorf AG, Germany). Tissue homogenates were resuspended in 10 mL of Percoll (GE Healthcare, Uppsala, Sweden) 30% and gently laid over 3 mL of Percoll 70%. After centrifugation at 18 °C, 950 × *g* for 20 min with centrifuge breaks turned off, the ring containing mononuclear cells was collected and washed twice with HBSS at 18 °C, 450 × g for 7 min. Cells were adjusted to 5 × 10^5^ cells in 0.5 mL of RPMI medium supplemented with 10% heat-inactivated fetal calf serum (Gibco, United States), 4 mM of L-glutamine (Sigma-Aldrich), 1% of sodium pyruvate (Sigma-Aldrich), 1% of nonessential amino acids (Sigma-Aldrich) and 0.1% antibiotic/antimycotic (Sigma-Aldrich). CNS cells were distributed in 24-well plate, stimulated with MOG_35-55_ (50 µg/mL) and incubated at 37 °C in a humidified incubator containing 5% CO_2_. After 48 h incubation, supernatants were stored at -20 °C for cytokine quantification.

### Gut explant immersion cultures

Cytokine production by immune cells from lamina propria was checked by using gut explant immersion cultures which were done with small fragments from jejunum and ileum. This approach was adapted from Bareiss et al.^85^ and Randall et al.^86^. Briefly, the entire jejunum and ileum were aseptically removed and placed in sterile Petri dishes with cold HBSS containing 1% of antibiotic/antimycotic (Sigma-Aldrich). The cleaned sections (4 -5 mm), devoid of faeces and Peyer's patches, were sectioned longitudinally to expose the mucosal surface and placed in a 48-well plate. Each well contained two explants immersed in 0.5 ml of RPMI 1640 medium supplemented with 10% heat-inactivated fetal calf serum (Gibco, United States), 4 mM of l-glutamine (Sigma-Aldrich), 1% of sodium pyruvate (Sigma-Aldrich), 1% of nonessential amino acids (Sigma-Aldrich) and 2% antibiotic/antimycotic (Sigma-Aldrich). The cultures were incubated in a humidified incubator at 37 °C and 5% CO_2_ for up to 6 h after which the supernatants were stored at − 20 °C for further cytokine quantification.

### RT-qPCR analysis

RNA from lumbar spinal cord and fragments of the small intestine (jejunum and ileum) were extracted with TRIzol reagent (Life Technologies, Carlsbad, CA, United States). For cDNA synthesis, 1000 ng of RNA was converted to cDNA using High Capacity cDNA Reverse Transcription kit (Life Technologies) following manufacturer’s instructions. Expression of *TAU* (Mm00521988_m1), *Tbx21* (Mm00450960_m1), *Rorc* (Mm01261022_m1), *Foxp3* (Mm00475162_m1), *Tgf* (Mm01178820 m1), *Clec7* (Mm01183349_m1), *Tlr*2 (Mm00442346_m1), *Tlr4* (Mm00445273_m1) and *Ido* (Mm00492590 m1) target genes was analyzed and normalized in comparison to the reference gene *GAPDH* (Mm99999915_m1), which show similar expression among groups. Real Time PCR was performed using TaqMan Gene Expression Assays (Applied Biosystems, Foster City, CA, United States) according to manufacturer’s instructions in ABI 7300 equipment (Applied Biosystems). Data were analyzed in SDS Software System 7300 and mRNA relative expression was determined using the equation 2 ^− ΔΔCt^^87^. This equation is based on the difference in the cycle threshold (ΔCt) for target and reference genes, divided by the Ct for the calibrator (ΔΔCt); considering the mean of control (non-EAE and non-supplemented mice) group as the calibrator.

### Cytokine quantification

Cytokine production by the spleen and CNS cell cultures was evaluated by cytometric bead array (CBA) in supernatants using Mouse Th1/Th2/Th17 Cytokine Kit (BD Biosciences, San Diego, CA, USA) according to manufacturer’s instructions. Data acquisition was performed using a FACS Canto II flow cytometer (BD Biosciences) from Institute of Biosciences (UNESP, Botucatu, SP, Brazil) and the data were analyzed with FCAP Array 3.0 (Soft Flow Inc., St. Louis Park, MN, USA). Cytokine production by gut explant immersion cultures was evaluated by enzyme-linked immunosorbent assay (ELISA), using Mouse DuoSet ELISA (R&D Systems, Minneapolis, MN, USA), according to the instructions of the manufacturer.

### Flow cytometry

The percentage of regulatory T (CD3^+^CD4^+^CD25^+^FoxP3^+^) and DC (CD11c^+^MHCII^High^) were evaluated in total cells from the MLN by flow cytometry. Samples were incubated with PerCP-Cy5.5 labelled anti-mouse CD3 (clone 145-2C11), PE-Cy7 labelled anti-mouse CD4 (clone GK1.5) and APC labelled anti-mouse CD25 (clone PC61.5) for T cell subsets panel or with PerCP-Cy5.5 labelled anti-mouse CD11b (clone M1/70), APC-Cy7 labelled anti-mouse CD11c (clone N418), APC labelled anti-mouse MHCII (clone M5/114.15.2), PE labelled anti-mouse CD103 (clone 2E7) and PE-Cy7 anti-mouse PD-L1 (clone MIH5) for DC panel during 30 min at 4 °C. Intracellular Foxp3 transcription factor was detected using anti-mouse/rat Foxp3 Staining Set PE (FJK-16s) (eBiosciences, San Diego, CA, USA) according to manufacturer's instructions. After staining, the cells were washed, resuspended in flow cytometry buffer and fixed in paraformaldehyde 1%. Flow cytometry was performed using a FACS Canto II (BD Biosciences) from Institute of Biosciences (UNESP, Botucatu, SP, Brazil), the compensation of acquisition data was performed using OneComp eBeads Compensation Beads (eBiosciences) and data were analyzed with FlowJo software (BD Biosciences). Gating strategy for T cell and DC subsets was showed in Supplementary Fig. 2.

### Microbiota analysis

Faecal DNA was extracted with ZymoBIOMICS DNA Miniprep Kit (Zymo Research, Irvine, CA, USA) according to manufacturer’s instructions. Each DNA sample was subsequently used for 16S amplification using appropriate negative (UltraClean DNA-free PCR water; MO BIO Laboratories, Inc., Carlsbad, CA, USA) and positive (ZymoBIOMICS Microbial Community Standards, Zymo Research) controls. Metagenomic DNA was amplified using the V4 region of the 16S rRNA gene that was amplified with region-specific primers that included the Illumina flowcell adapter sequences. The reverse amplification primer also contained a twelve-base barcode sequence that supports pooling of different samples for Illumina sequencing. After cluster formation, the amplicons were sequenced with custom primers. These sequencing primers were designed to be complementary to the V4 amplification primers to avoid sequencing of the primers, and the barcode was read using a third sequencing primer in an additional cycle. Amplification primers (forward GTGCCAGCMGCCGCGGTAA and reverse GGACTACHVGGGTWTCTAAT) were adapted from Caporaso et al.^88^ protocol to include nine extra bases in the adapter region of the forward amplification primer that supported paired-end sequencing. Libraries were normalized and pooled to 4 nM based on qPCR values. Pooled samples were denatured and diluted to a final concentration of 10 pM with a 20% PhiX (Illumina) control. Sequencing was performed using the MiSeq Reagent Kit V3 in the Illumina MiSeq System. All 47 samples were multiplexed and sequenced on the MiSeq using 2 × 150 bp paired-end sequencing. Analysis was performed with QIIME2 version 2020.2^89^. Briefly, low quality sequences and chimeras were discarded with DADA2 pipeline with standard parameters. Alfa rarefaction and beta diversity were performed with a sampling of 100,000 reads from each sample. Taxonomy was assigned in QIIME against the GreenGenes ribosomal RNA gene database (GreenGenes 13_8 99% OTUs from 515F/806R region).

### Statistical analysis

With the exception of the microbiota, normality of data was analyzed by Shapiro–Wilk test and significant outliers were determined by Grubbs' test. Comparisons between three groups were made by One-Way ANOVA or Two-Way ANOVA multiple comparisons followed by Tukey’s multiple comparisons test or Holm-Sidak's multiple comparisons test and the disease prevalence was analyzed by Log-rank (Mantel-Cox) test. Data analysis and figures were made in GraphPad Prism 7 (GraphPad Software Inc., San Diego, California, United States) and values of *p* < 0.05 were considered statistically significant. Permutational multivariate analysis of variance (PERMANOVA) tests of the gut microbiota on the unweighted UniFrac distances. Alpha diversity indices were calculated using PAST 3.26 software (https://www.softpedia.com/get/Science-CAD/PAST.shtml).

## Supplementary Information

Supplementary Figures.

Supplementary Table.

## References

[1] Wootla B, Eriguchi M, Rodriguez M (2012). Is multiple sclerosis an autoimmune disease?. Autoimmune Dis..

[2] Legroux L, Arbour N (2016). Multiple sclerosis and T lymphocytes: an entangled story T lymphocytes: key cells of the adaptive immune responses. J. Neuroimmune Pharmacol..

[3] Robinson, A. P., Harp, C. T., Noronha, A. & Miller, S. D. NIH Public Access. 173–189 (2014) 10.1016/B978-0-444-52001-2.00008-X.The.

[4] Gajofatto A, Benedetti MD (2015). Treatment strategies for multiple sclerosis: when to start, when to change, when to stop?. World J. Clin. Cases.

[5] Dendrou CA, Fugger L (2014). Previews please mind the gap: axonal transport deficits in multiple sclerosis neurodegeneration. Neuron.

[6] Tanasescu, R., Ionete, C. & Constantinescu, C. S. Advances in the Treatment of Relapsing—Remitting Multiple Sclerosis (2014). 10.4103/2319-4170.130440 24732658

[7] Alonso R (2018). Changes in the multiple sclerosis treatment paradigm. What do we do now and what were we doing before?. J. Clin. Neurol..

[8] Wingerchuk DM, Carter JL (2014). Multiple sclerosis: current and emerging disease-modifying therapies and treatment strategies. Mayo Clin. Proc..

[9] Rommer PS, Zettl UK (2018). Expert opinion on pharmacotherapy managing the side effects of multiple sclerosis therapy: pharmacotherapy options for patients. Expert Opin. Pharmacother..

[10] Yadav, V. *et al.* Summary of evidence-based guideline: complementary and alternative medicine in multiple sclerosis Report of the Guideline Development Subcommittee of the American Academy of Neurology. 1083–1092 (2014). 10.1212/WNL.0000000000000250 PMC3962995 24663230

[11] Claflin SB, Van Der Mei IAF, Taylor BV (2017). Complementary and alternative treatments of multiple sclerosis: a review of the evidence from 2001 to 2016. J. Neurol. Neurosurg. Psychiatry.

[12] Wilmot VA, Swank RL (1952). The influence of low-fat diet on blood lipid levels in health and in multiple sclerosis. Am. J. Med. Sci..

[13] Swank RL (1953). Treatment of multiple sclerosis with low-fat diet. AMA Arch. Neurol. Psychiatry.

[14] Chenard CA, Rubenstein LM, Snetselaar LG, Wahls TL (2019). Nutrient composition comparison between the low saturated fat swank diet for multiple sclerosis and healthy U.S.-style eating pattern. Nutrients.

[15] Leong EM (2009). Complementary and alternative medicines and dietary interventions in multiple sclerosis: what is being used in South Australia and why?. Complement Ther. Med..

[16] Beckett JM, Bird M, Pittaway JK, Ahuja KDK (2018). Diet and multiple sclerosis: scoping review of web-based recommendations. Interact. J. Med. Res..

[17] Kirby TO (2018). The Gut microbiome in multiple sclerosis: a potential therapeutic avenue. J. Med. Sci..

[18] Riccio P, Rossano R (2018). Diet, gut microbiota, and vitamins D + A in multiple sclerosis. Neurotherapeutics.

[19] Gagliardi A (2018). Rebuilding the Gut microbiota ecosystem. Int. J. Environ. Res. Public Health.

[20] Takata K (2015). Dietary yeasts reduce inflammation in central nerve system via microflora. Ann. Clin. Transl. Neurol..

[21] He B (2019). *Lactobacillus reuteri* reduces the severity of experimental autoimmune encephalomyelitis in mice by modulating gut microbiota. Front. Immunol..

[22] Pericolini E (2017). Therapeutic activity of a *Saccharomyces cerevisiae*-based probiotic and inactivated whole yeast on vaginal candidiasis. Virulence.

[23] Roussel C (2018). Anti-infectious properties of the probiotic *Saccharomyces cerevisiae* CNCM I-3856 on enterotoxigenic *E. coli* (ETEC) strain H10407. Appl. Microbiol. Biotechnol..

[24] Pan X, Chen F, Wu T, Tang H, Zhao Z (2009). Prebiotic oligosaccharides change the concentrations of short-chain fatty acids and the microbial population of mouse bowel. J. Zhejiang Univ. Sci. B.

[25] Miest JJ, Arndt C, Adamek M, Steinhagen D, Reusch TBH (2016). Dietary β-glucan (MacroGard) enhances survival of first feeding turbot (*Scophthalmus maximus*) larvae by altering immunity, metabolism and microbiota. Fish Shellfish Immunol..

[26] Li H (2013). Low dose zymosan ameliorates both chronic and relapsing experimental autoimmune encephalomyelitis. J. Neuroimmunol..

[27] Duntas LH, Benvenga S (2015). Selenium: an element for life. Endocrine.

[28] Soni C (2019). Selenium supplementation suppresses immunological and serological features of lupus in B6.Sle1b mice. Autoimmunity.

[29] Zhai Q (2018). Effects of dietary selenium supplementation on intestinal barrier and immune responses associated with its modulation of gut microbiota. Environ. Sci. Technol. Lett..

[30] Cording S (2014). The intestinal micro-environment imprints stromal cells to promote efficient treg induction in gut-draining lymph nodes. Mucosal Immunol..

[31] Randolph GJ, Ivanov S, Zinselmeyer BH, Scallan JP (2017). The lymphatic system: integral roles in immunity. Annu. Rev. Immunol..

[32] Ducray HAG (2019). Yeast fermentate prebiotic improves intestinal barrier integrity during heat stress by modulation of the gut microbiota in rats. J. Appl. Microbiol..

[33] Duc D (2019). disrupting myelin-specific Th17 cell gut homing confers protection in an adoptive transfer experimental autoimmune encephalomyelitis. Cell Rep..

[34] Tofalo R (2019). The life and times of yeasts in traditional food fermentations. Crit. Rev. Food Sci. Nutr..

[35] Nandy SK, Srivastava RK (2018). A review on sustainable yeast biotechnological processes and applications. Microbiol. Res..

[36] Srivastava RK (2019). Yeast species mediated bioprocesses and bio-products for biotechnological application. J. Biotechnol. Biomed. Sci..

[37] Gaensly F, Wille GMFC, Brand D, Bonfim TMB (2011). Iron enriched *Saccharomyces cerevisiae* maintains its fermenting power and bakery properties. Ciência Tecnol. Aliment..

[38] Suhajda A, Janzs B, Pais I, Vereczkey G (2000). Trace elements preparation of selenium yeasts I. Preparation of selenium-enriched Saccharomyces cerevisiae. Prep. Se Yeast.

[39] Oraby MM, Allababidy T, Ramadan EM (2015). The bioavailability of selenium in *Saccharomyces cerevisiae*. Ann. Agric. Sci..

[40] Porto BAA (2019). Treatment with selenium-enriched *Saccharomyces cerevisiae* UFMG A-905 partially ameliorates mucositis induced by 5-fluorouracil in mice. Cancer Chemother. Pharmacol..

[41] Cardoso BR, Roberts BR, Bush AI, Hare DJ (2015). Selenium, selenoproteins and neurodegenerative diseases. Metallomics.

[42] Socha K (2014). Dietary habits and selenium, glutathione peroxidase and total antioxidant status in the serum of patients with relapsing-remitting multiple sclerosis. Nutr. J..

[43] Korpela H, Kinnunen E, Juntunen J, Kumpulainen J, Koskenvuo M (1989). Serum selenium concentration, glutathione peroxidase activity and lipid peroxides in a co-twin control study on multiple sclerosis. J. Neurol. Sci..

[44] Alizadeh A (2016). Comparison of serum Concentration of Se, Pb, Mg, Cu, Zn, between MS patients and healthy controls. Electron. Phys..

[45] Pîrşcoveanu DFV (2017). Tau protein in neurodegenerative diseases—a review. Rom. J. Morphol. Embryol..

[46] Weinger JG (2012). Mice devoid of tau have increased susceptibility to neuronal damage in myelin oligodendrocyte glycoprotein-induced experimental autoimmune encephalomyelitis. J. Neuropathol. Exp. Neurol..

[47] Jaworski J, Psujek M, Janczarek M, Szczerbo-Trojanowska M, Bartosik-Psujek H (2012). Total-tau in cerebrospinal fluid of patients with multiple sclerosis decreases in secondary progressive stage of disease and reflects degree of brain atrophy. Ups. J. Med. Sci..

[48] Kosehasanogullari G, Ozakbas S, Idiman E (2015). Tau protein levels in the cerebrospinal fluid of the patients with multiple sclerosis in an attack period: Low levels of tau protein may have significance, too. Clin. Neurol. Neurosurg..

[49] Luo J (2007). Glia-dependent TGF-β signaling, acting independently of the TH17 pathway, is critical for initiation of murine autoimmune encephalomyelitis. J. Clin. Invest..

[50] Danikowski KM, Jayaraman S, Prabhakar BS (2017). Regulatory T cells in multiple sclerosis and myasthenia gravis. J. Neuroinflamm..

[51] Baecher-Allan C, Kaskow BJ, Weiner HL (2018). Multiple sclerosis: mechanisms and immunotherapy. Neuron.

[52] Sage PT (2018). Dendritic cell PD-L1 limits autoimmunity and follicular T cell differentiation and function. J. Immunol..

[53] Shiokawa A, Kotaki R, Takano T, Nakajima-Adachi H, Hachimura S (2017). Mesenteric lymph node CD11b− CD103+ PD-L1 High dendritic cells highly induce regulatory T cells. Immunology.

[54] Xue H (2010). Selenium upregulates CD4 + CD25 + regulatory T cells in iodine-induced autoimmune thyroiditis model of. Endocr. J..

[55] Nettleford SK, Prabhu KS (2018). Selenium and selenoproteins in gut inflammation—a review. Antioxidants.

[56] Sonnenburg ED (2016). Diet-induced extinctions in the gut microbiota compound over generations. Nature.

[57] Kriss M, Hazleton KZ, Nusbacher NM, Martin CG, Lozupone CA (2018). Low diversity gut microbiota dysbiosis: drivers, functional implications and recovery. Curr. Opin. Microbiol..

[58] Bello MGD, Knight R, Gilbert JA, Blaser MJ (2018). Preserving microbial diversity. Science.

[59] Lozupone CA, Stombaugh JI, Gordon JI, Jansson JK, Knight R (2012). Diversity, stability and resilience of the human gut microbiota. Nature.

[60] Jangi S (2016). Alterations of the human gut microbiome in multiple sclerosis. Nat. Commun..

[61] Shahi SK, Freedman SN, Mangalam AK (2017). Gut microbiome in multiple sclerosis: the players involved and the roles they play. Gut Microbes.

[62] Chu F (2018). Gut microbiota in multiple sclerosis and experimental autoimmune encephalomyelitis: current applications and future perspectives. Mediators Inflamm..

[63] Freedman SN, Shahi SK, Mangalam AK (2018). The, “gut feeling”: breaking down the role of gut microbiome in multiple sclerosis. Neurotherapeutics.

[64] Gandy KAO, Zhang J, Nagarkatti P, Nagarkatti M (2019). The role of gut microbiota in shaping the relapse-remitting and chronic-progressive forms of multiple sclerosis in mouse models. Sci. Rep..

[65] Hakansson A, Molin G (2011). Gut microbiota and inflammation. Nutrients.

[66] Wang K (2017). Lactobacillus casei regulates differentiation of th17/treg cells to reduce intestinal inflammation in mice. Can. J. Vet. Res..

[67] He B, Hoang TK, Tran DQ, Rhoads JM, Liu Y (2017). Adenosine A2A receptor deletion blocks the beneficial effects of *Lactobacillus reuteri* in regulatory T-Deficient scurfy mice. Front. Immunol..

[68] Atarashi K (2013). Treg induction by a rationally selected mixture of Clostridia strains from the human microbiota. Nature.

[69] Nagano Y, Itoh K, Honda K (2012). The induction of Treg cells by gut-indigenous Clostridium. Curr. Opin. Immunol..

[70] Macpherson AJ, Smith K (2006). Mesenteric lymph nodes at the center of immune anatomy. J. Exp. Med..

[71] Kobayashi H (2004). In situ demonstration of dendritic cell migration from rat intestine to mesenteric lymph nodes: relationships to maturation and role of chemokines. J. Leukoc. Biol..

[72] Nouri M, Bredberg A, Weström B, Lavasani S (2014). Intestinal barrier dysfunction develops at the onset of experimental autoimmune encephalomyelitis, and can be induced by adoptive transfer of auto-reactive T cells. PLoS ONE.

[73] Price AE (2018). A map of toll-like receptor expression in the intestinal epithelium reveals distinct spatial, cell type-specific, and temporal patterns. Immunity.

[74] Reynolds JM, Martinez GJ, Chung Y, Dong C (2012). Toll-like receptor 4 signaling in T cells promotes autoimmune inflammation. Proc. Natl. Acad. Sci. U. S. A..

[75] Esplugues E (2011). Control of TH17 cells occurs in the small intestine. Nature.

[76] Berer K, Krishnamoorthy G (2014). Microbial view of central nervous system autoimmunity. FEBS Lett..

[77] Rhee SH, Pothoulakis C, Mayer EA (2009). Principles and clinical implications of the brain–gut–enteric microbiota axis. Nat. Rev. Gastroenterol. Hepatol..

[78] Montiel-Castro AJ, Gonzalez-Cervantes RM, Bravo-Ruiseco G, Pacheco-Lopez G (2013). The microbiota-gut-brain axis: Neurobehavioral correlates, health and sociality. Front. Integr. Neurosci..

[79] Marietta E, Horwath I, Taneja V (2018). Microbiome, immunomodulation, and the neuronal system. Neurotherapeutics.

[80] Kopp TI, Outzen M, Olsen A, Vogel U, Ravn-Haren G (2018). Genetic polymorphism in selenoprotein P modifies the response to selenium-rich foods on blood levels of selenium and selenoprotein P in a randomized dietary intervention study in Danes. Genes Nutr..

[81] Reeves PG, Nielsen FH, Fahey GC (1993). AIN-93 purified diets for laboratory rodents: Final report of the American Institute of Nutrition ad hoc writing committee on the reformulation of the AIN-76A rodent diet. J. Nutr..

[82] Brocke S, Gijbels K, Steinman L (1994). Experimental Autoimmune Encephalomyelitis in the Mouse.

[83] Fraga-Silva TFC (2016). Tolerogenic vaccination with MOG/VitD overcomes aggravating effect of *C. albicans* in experimental encephalomyelitis. CNS Neurosci. Ther..

[84] Vieira AT (2012). Treatment with Selemax, a selenium-enriched yeast, ameliorates experimental arthritis in rats and mice. Br. J. Nutr..

[85] Bareiss PM (2008). Organotypical tissue cultures from adult murine colon as an in vitro model of intestinal mucosa. Histochem. Cell Biol..

[86] Randall KJ, Turton J, Foster JR (2011). Explant culture of gastrointestinal tissue: a review of methods and applications. Cell Biol. Toxicol..

[87] Livak KJ, Schmittgen TD (2001). Analysis of relative gene expression data using real-time quantitative PCR and the 2(-Delta Delta C(T)) Method. Methods.

[88] Caporaso JG (2010). QIIME allows analysis of high-throughput community sequencing data. Nat. Methods.

[89] Bolyen E (2019). Reproducible, interactive, scalable and extensible microbiome data science using QIIME 2. Nat. Biotechnol..

