# Supplementary material for: Selenization of S. cerevisiae increases its protective potential in experimental autoimmune encephalomyelitis by triggering an intestinal immunomodulatory loop
Source: Sci Rep. 2020 Dec 17;10:22190. doi: 10.1038/s41598-020-79102-7 (PMC7746691; doi:10.1038/s41598-020-79102-7)
Supplement: Supplementary file 1 — Supplementary Figures. [file 41598_2020_79102_MOESM1_ESM.docx]

**Selenization of *S. cerevisiae* increases its protective potential in experimental autoimmune encephalomyelitis by triggering an intestinal immunomodulatory loop**

Thais Fernanda de Campos Fraga-Silva^†*^, Luiza Ayumi Nishiyama Mimura^‡^, Larissa Ragozo Cardoso de Oliveira^‡^, Juliana Helena dos Santos Toledo^‡^, Patrícia Aparecida Borim^†^, Sofia Fernanda Gonçalvez Zorzella-Pezavento^‡^, Diego Peres Alonso^§^, Paulo Eduardo Martins Ribolla^§^, Carlos Alberto Ferreira de Oliveira^#^, Denise Morais da Fonseca^&^, Eduardo J. Villablanca^+^, Alexandrina Sartori†‡

^†^ Botucatu Medical School, São Paulo State University (UNESP), Botucatu, Brazil;

^‡^ Institute of Biosciences, São Paulo State University (UNESP), Botucatu, Brazil;

^§^ Institute of Biotechnology (IBTEC), São Paulo State University (UNESP), Botucatu, Brazil.

^#^ Biorigin, Zilor, Lençóis Paulista, Brazil.

^&^ Institute of Biomedical Sciences, University of São Paulo (USP), São Paulo, Brazil.

^+^ Immunology and Allergy Unit, Department of Medicine, Solna, Karolinska Institutet and University Hospital, Stockholm, Sweden.

* Corresponding author: Thais F. C. Fraga-Silva (thaisfragasilva@gmail.com).

**Selenized yeast decreases encephalomyelitis severity**


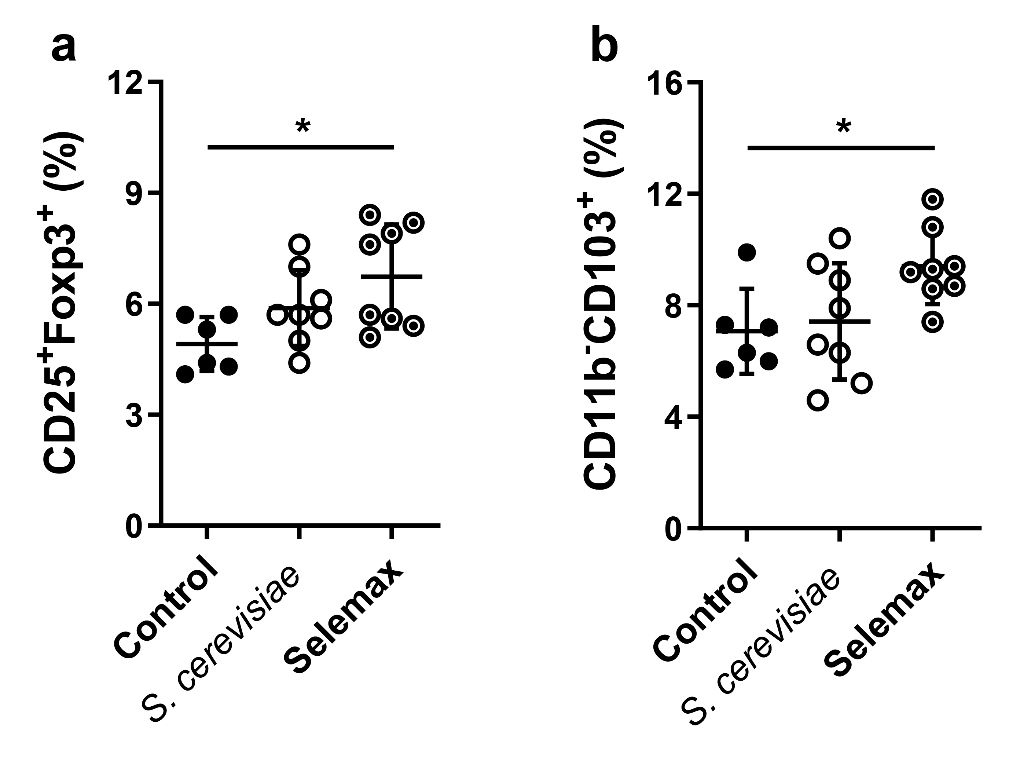


**Supplementary Figure 1. Effect of *S. cerevisiae* and Selemax on the proportion of Tregs and tolerogenic DC in mesenteric lymph nodes from non-EAE mice.** Healthy mice (non-EAE mice) received 14 oral doses of *S. cerevisiae* or Selemax. Total cells from mesenteric lymph nodes were collected and analyzed by flow cytometry to determine the proportion of T regulatory - Treg (CD25^+^Foxp3^+^) (a) and tolerogenic DC (CD11b^-^CD103^+^) (b) subsets. Statistical analysis was performed by t-test. All data were expressed as the mean ± SD and statistical differences were represented by *p<0.05 and **p<0.01. Two independent experiments were combined, n = 6-8 mice/group. The graphs were created using GraphPad Prism v.8.0.2 and image was modified with Adobe Photoshop v.22.0.0.


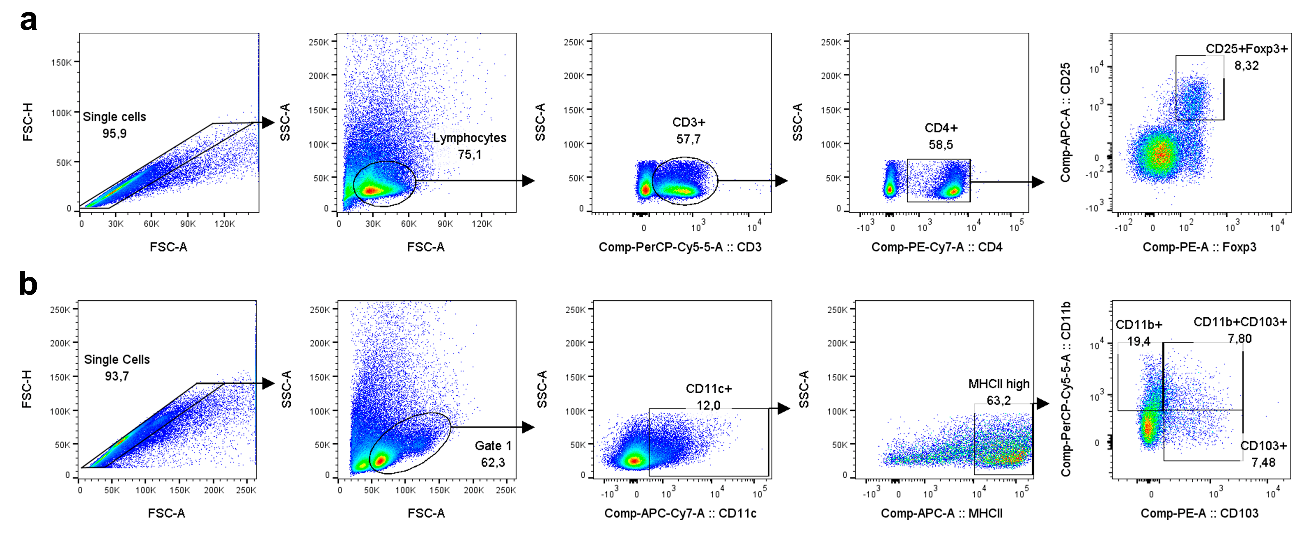


**Supplementary Figure 2. Flow cytometric gating strategy for T cell and DC subsets.** Total cells from inguinal and mesenteric lymph nodes were collected and analyzed by flow cytometry. The proportion of T cell (CD3^+^) subsets was evaluated in single cells (FSC-H vs FSC-A) with lymphocyte morphology by forward versus side scatter (FSC vs SSC) gating. The proportion of T helper cells – Th (CD3^+^CD4^+^) was gating to assess the proportion of T regulatory - Treg (CD25^+^Foxp3^+^) (a). The proportion of dendritic cells – DC (CD11c^+^MHCII^High^) subsets was evaluated in single cells (FSC-H vs FSC-A) with no defined morphology by forward versus side scatter (FSC vs SSC) gate 1. The proportions of CD11b^+^CD103^-^, CD11b^+^CD103^+^ and CD11b^-^CD103^+^ DC subsets were assessed in total DC. The mean fluorescence intensity of PD-L1 was assess only in total tolerogenic DC (CD11b^-^CD103^+^) subset (b). Non-stained cells were used to define gating strategy. Flow cytometry images were created using FlowJo 10.7.1 and image was modified with Adobe Photoshop v.22.0.0.
