# Supplementary material for: Selenization of S. cerevisiae increases its protective potential in experimental autoimmune encephalomyelitis by triggering an intestinal immunomodulatory loop
Source: Sci Rep. 2020 Dec 17;10:22190. doi: 10.1038/s41598-020-79102-7 (PMC7746691; doi:10.1038/s41598-020-79102-7)
Supplement: Supplementary file 2 — Supplementary Table. [file 41598_2020_79102_MOESM2_ESM.docx]

**Selenization of *S. cerevisiae* increases its protective potential in experimental autoimmune encephalomyelitis by triggering an intestinal immunomodulatory loop**

Thais Fernanda de Campos Fraga-Silva^†*^, Luiza Ayumi Nishiyama Mimura^‡^, Larissa Ragozo Cardoso de Oliveira^‡^, Juliana Helena dos Santos Toledo^‡^, Patrícia Aparecida Borim^†^, Sofia Fernanda Gonçalvez Zorzella-Pezavento^‡^, Diego Peres Alonso^§^, Paulo Eduardo Martins Ribolla^§^, Carlos Alberto Ferreira de Oliveira^#^, Denise Morais da Fonseca^&^, Eduardo J. Villablanca^+^, Alexandrina Sartori†‡

^†^ Botucatu Medical School, São Paulo State University (UNESP), Botucatu, Brazil;

^‡^ Institute of Biosciences, São Paulo State University (UNESP), Botucatu, Brazil;

^§^ Institute of Biotechnology (IBTEC), São Paulo State University (UNESP), Botucatu, Brazil.

^#^ Biorigin, Zilor, Lençóis Paulista, Brazil.

^&^ Institute of Biomedical Sciences, University of São Paulo (USP), São Paulo, Brazil.

^+^ Immunology and Allergy Unit, Department of Medicine, Solna, Karolinska Institutet and University Hospital, Stockholm, Sweden.

* Corresponding author: Thais F. C. Fraga-Silva (thaisfragasilva@gmail.com).

**Selenized yeast decreases encephalomyelitis severity**

**Supplementary Table 1. Experimental design.**

| **Local** | **Sample** | **Method** | **Parameter** | **Experiment** |
| --- | --- | --- | --- | --- |
| **Blood** | Serum | Biochemistry | Total selenium, renal and hepatic function parameters | 2 and 3 |
| **CNS** | Lumbar spinal cord | Histopathology | Inflammation and demyelination | 4 |
|  |  | RT-qPCR | Gene expression | 2 and 3 |
|  | Brain + spinal cord  (cervical and thoracic) | Cell culture | Cytokine production | 3 and 4 |
| **Spleen** | Total cells | Cell culture | Cytokine production | 3 and 4 |
| **Inguinal lymph nodes** | Total cells | Flow cytometry | T cells subsets | 1 and 4 |
| **Mesenteric lymph nodes** | Total cells | Flow cytometry | T cells and DC subsets | 2 and 3 |
| **Small intestine** | Jejunum and ileum | RT-qPCR | Gene expression | 3 and 4 |
|  |  | Explant culture | Cytokine production | 3 and 4 |
|  |  | Histopathology | Inflammation | 4 |
| **Feces** | Fresh stool | Next-Generation Sequencing | Microbiome | 2 and 3 |
